# Comparative field evaluation of combinations of long-lasting insecticide treated nets and indoor residual spraying, relative to either method alone, for malaria prevention in an area where the main vector is *Anopheles arabiensis*

**DOI:** 10.1186/1756-3305-6-46

**Published:** 2013-02-22

**Authors:** Fredros O Okumu, Edgar Mbeyela, Godfrey Lingamba, Jason Moore, Alex J Ntamatungiro, Deo R Kavishe, Michael G Kenward, Elizabeth Turner, Lena M Lorenz, Sarah J Moore

**Affiliations:** 1 Environmental Health and Ecological Sciences Thematic Group, Ifakara Health Institute, Ifakara, Tanzania; 2 Department of Disease Control, London School of Hygiene and Tropical Medicine, London, UK; 3 Department of Medical Statistics, London School of Hygiene and Tropical Medicine, London, UK

## Abstract

**Background:**

Long-lasting insecticidal nets (LLINs) and indoor residual spraying (IRS) are commonly used together in the same households to improve malaria control despite inconsistent evidence on whether such combinations actually offer better protection than nets alone or IRS alone.

**Methods:**

Comparative tests were conducted using experimental huts fitted with LLINs, untreated nets, IRS plus untreated nets, or combinations of LLINs and IRS, in an area where *Anopheles arabiensis* is the predominant malaria vector species. Three LLIN types, Olyset®, PermaNet 2.0® and Icon Life® nets and three IRS treatments, pirimiphos-methyl, DDT, and lambda cyhalothrin, were used singly or in combinations. We compared, number of mosquitoes entering huts, proportion and number killed, proportions prevented from blood-feeding, time when mosquitoes exited the huts, and proportions caught exiting. The tests were done for four months in dry season and another six months in wet season, each time using new intact nets.

**Results:**

All the net types, used with or without IRS, prevented >99% of indoor mosquito bites. Adding PermaNet 2.0® and Icon Life®, but not Olyset® nets into huts with any IRS increased mortality of malaria vectors relative to IRS alone. However, of all IRS treatments, only pirimiphos-methyl significantly increased vector mortality relative to LLINs alone, though this increase was modest. Overall, median mortality of *An. arabiensis* caught in huts with any of the treatments did not exceed 29%. No treatment reduced entry of the vectors into huts, except for marginal reductions due to PermaNet 2.0® nets and DDT. More than 95% of all mosquitoes were caught in exit traps rather than inside huts.

**Conclusions:**

Where the main malaria vector is *An. arabiensis*, adding IRS into houses with intact pyrethroid LLINs does not enhance house-hold level protection except where the IRS employs non-pyrethroid insecticides such as pirimiphos-methyl, which can confer modest enhancements. In contrast, adding intact bednets onto IRS enhances protection by preventing mosquito blood-feeding (even if the nets are non-insecticidal) and by slightly increasing mosquito mortality (in case of LLINs). The primary mode of action of intact LLINs against *An. arabiensis* is clearly bite prevention rather than insecticidal activity. Therefore, where resources are limited, priority should be to ensure that everyone at risk consistently uses LLINs and that the nets are regularly replaced before being excessively torn. Measures that maximize bite prevention (e.g. proper net sizes to effectively cover sleeping spaces, stronger net fibres that resist tears and burns and net use practices that preserve net longevity), should be emphasized.

## Background

Most of the recent and historical reductions in malaria cases have been attributed to insecticide treated nets (ITNs), and indoor residual insecticide spraying (IRS) [1-9] These methods are currently supported by an exemplary level of public and political goodwill, and are mostly used alongside other strategies such as prompt and accurate diagnosis [10-12], treatment with artemisinin based medicines [3,5,13-16], and intermittent preventive treatment (IPT) [17,18], all of which are also significantly contributing to the gains.

Although long lasting insecticidal nets (LLINs) are designed as stand-alone vector control tools, there are several instances where they are supplemented with IRS in the same houses, often with the aim of achieving greater health benefits. In an earlier review article [19], where published records of combining LLINs with IRS were explored, it was noted that other than a small amount of indirect field evidence [20-23], and an assortment of theoretical simulations [24-26] suggesting added advantages of simultaneous use, over either LLINs alone or IRS alone, there had been no studies that explicitly determined whether combining LLINs with IRS in the same households would have synergistic or redundant effects [19]. Since that review, at least one study, conducted in Benin, has shown that combinations of deltamethrin-based LLINs with chlorfenapyr, a pyrole insecticide, have potential to not only provide additional protection relative to the components singly, but also that such combinations can be effective against insecticide resistant vector populations [23]. Elsewhere, a non-randomized prospective study embedded in a programmatic anti-malaria operation in western Kenya concluded that the protective efficacy of ITNs combined with IRS was 62% greater than ITNs alone [27]. The authors of this work, however, also suggested the need for confirmation of their results through randomised controlled trials and also the need for a cost effectiveness analysis of LLIN-IRS combinations [27]. Following that study, a recent randomised controlled trial covering 58 villages in Benin has shown no significant reduction in malaria morbidity, infection rates and transmission intensities in communities using combinations of LLINs with either IRS or insecticide treated plastic wall sheeting, as compared to villages using only LLINs [28]. Though this particular trial was conducted in an area with high pyrethroid resistance, the insecticide used for IRS was bendiocarb, which had remained effective in Benin despite the high levels of pyrethroid resistance [29]. Thus, some added advantages of the LLIN/IRS combinations relative to LLINs alone would have been expected.

There are also a number of theoretical justifications for combining LLINs with IRS, namely: 1) the fact that nets may get torn over time, 2) IRS may decay with time, 3) people with nets may not always use those nets correctly and consistently, and 4) some IRS/LLIN combinations could potentially delay the onset and spread of insecticide resistance. All of these justifications further emphasize the urgent need to optimise current LLIN-IRS strategies. It has previously been suggested that future simultaneous insecticide applications should involve insecticides with different modes of action, rather than just the pyrethroid-based LLINs [30]. However, overall epidemiological outcomes of any LLIN/IRS co-applications will depend also on the extent of intervention coverage in target communities, baseline epidemiological conditions and behaviour of local malaria vectors [19]. Thus, a series of studies, including entomological, epidemiological, and cost-effectiveness studies, should be conducted to generate direct evidence for or against these combinations under local conditions, so as to guide any programmatic implementation.

The purpose of the current study was therefore to contribute essential empirical evidence on protective efficacy of LLIN/IRS combinations, particularly in endemic communities where residual malaria vector populations consist predominantly of *Anopheles arabiensis*. Various indicators of protection were assessed inside experimental huts [31] where both LLINs and IRS were used, and compared to similar observations in huts where either LLINs alone, IRS alone or non-insecticidal nets were used. Since WHO has approved a variety of LLINs (with different pyrethroids as active ingredients), and also several classes of insecticides for IRS [32], this study involved multiple combinations of net types and IRS insecticide classes, providing a unique opportunity to not only compare the insecticidal applications, but to also select the most appropriate candidates for optimal combination.

## Methods

### Study area

The study was conducted in Lupiro village (8.385^o^S and 36.670^o^E) in Ulanga District, south eastern Tanzania. The village lies 270 m above sea level on the Kilombero river valley, and is 26 km south of Ifakara town, where Ifakara Health Institute (IHI) is located. It borders many small contiguous and perennially swampy rice fields to the northern and eastern sides. The annual rainfall is 1200-1800 mm, while temperatures range between 20°C and 33°C. Composition of malaria vector populations (which previously included a mixture of *Anopheles gambiae sensu stricto, Anopheles arabiensis* and *Anopheles funestus* s.s.) has shifted dramatically in recent years, most likely because of high ITN coverage [33], so that today, the most abundant vector is *Anopheles arabiensis*, constituting > 95% of the *An. gambiae* complex species [34,35], followed by *An. funestus*. The *An. arabiensis* and *An. funestus* species are now the main contributors to malaria transmission in the area (Kaindoa *et al.,* Unpublished). WHO insecticide susceptibility tests conducted at the time of this study [36] showed that the *An. arabiensis* in the study area were 100% susceptible to DDT, but had slightly reduced susceptibility to the main pyrethroids, i.e. deltamethrin (95.8%), lambda cyhalothrin (90.2%) and permethrin (95.2%).

### LLINs and IRS compounds

Four net types (three LLINs and one non-insecticidal net) and three IRS insecticides of different classes (an organochloride, a synthetic pyrethroid, and an organophosphate) were used. The LLINs included Olyset® nets (manufactured by A-Z, Tanzania), PermaNet 2.0® nets (Vestergaard Frandsen, Switzerland) and ICON Life® nets (supplied by Syngenta, Switzerland), which has similar specifications as the one marketed under the brand name, NetProtect® (Bestnet Europe Ltd, Denmark) [37]. Olyset® nets are polyethylene (150 denier), impregnated during manufacture with synthetic permethrin at 2% w/w (equivalent to 1000 mg of active ingredient/m^2^). PermaNet 2.0® is 100%-polyester (100 denier), coated with 55-62 mg of synthetic deltamethrin/m^2^, resulting in insecticide concentrations of approximately 0.14% w/w, depending on mesh size. Icon Life® is also polyethylene (118 denier), impregnated during manufacture with synthetic deltamethrin at 0.2% w/w (approximately 65-79 mg of active ingredient/m^2^ depending on mesh size).

The IRS chemicals included: 1) an organochloride, 75% pure DDT wettable powder (AVIMA, South Africa), sprayed at 2 g/m^2^ concentration of the active ingredient (a.i), 2) a synthetic pyrethroid, 10% capsule suspension of lambda-cyhalothrin, brand named Icon 10 CS (Syngenta, Switzerland), sprayed at 0.03 g/m^2^ a.i, and 3) an organophosphate, 50% emulsified concentrate of pirimiphos-methyl, also known as Actellic EC (Syngenta, Switzerland), sprayed at 2 g/m^2^ a.i. The IRS compounds and all the LLINs have either full or interim approval from WHO, and therefore represent a diversity of common insecticides currently applicable for vector control in Africa [31].

### Experimental huts and mosquito traps

We used an improved version of experimental huts, now commonly referred to as the Ifakara experimental huts. Full details of this hut design, and all entomological practices associated with its use, have been described elsewhere [31]. These experimental huts have similar average dimensions and shape as local village houses used in southern Tanzania. They have galvanized iron frames and corrugated iron roofs, overlaid with grass thatch to regulate temperatures. The undersides of the roofs are covered with ceilings made of traditionally-woven grass mats locally known as *mikeka.* The walls are constructed using canvas on the outside and are lined on the inside with removable wood panels that are coated with clay mud to simulate traditional mud walls. These mud panels and *mikeka* ceilings, form the insecticide-sprayed surfaces, which can be removed and incinerated at the end of each experiment, then replaced in readiness for any new tests. Each hut has one door, four windows and open eave spaces all round [31].

To study behavioural and physiological responses of mosquitoes in and around the experimental huts, each hut was fitted with interception traps as follows: eight exit traps were fitted on eave spaces (eave traps), and window traps were fitted onto all the windows, so as to catch mosquitoes when exiting the huts. The eave traps were interspersed, so that there were adequate spaces between them to allow mosquitoes to enter on all four sides of the huts. These open spaces were fitted with baffles, i.e. netting barriers facing the inside of the huts but slanting upwards at approximately the same angle as the roofs. The baffles allow mosquitoes to enter freely, but restrict exit of those mosquitoes through the same openings, i.e. once the mosquitoes were inside the huts, they could exit only through the spaces fitted with exit traps [31].

### Study design

Nine experimental huts were set up in a linear conformation, with 20 to 50 metres between any two huts, at the edge of the study village, such that the huts were between the main mosquito aquatic habitats (the contiguous set of small perennially swampy rice fields) and the human houses. For ease of reference, the huts were assigned numbers 1–9 starting with the northernmost to the southernmost hut. Each of the huts was fitted with two beds. Two male volunteers, aged between 18 and 35 years, were assigned to each hut for the duration of the study, to sleep under intact nets on each bed, each night; as baits but also to collect mosquitoes from the huts.

The huts were first stratified by identifying six of them for IRS treatment, (i.e. huts 1, 3, 5, 7, 8 and 9), and three to remain unsprayed (i.e. huts 2, 4, and 6). Each of the six IRS-designated huts was then randomly assigned to be sprayed with one of the three candidate insecticides for IRS, (such that there were two randomly assigned huts sprayed with each insecticide). The IRS was applied at WHO-approved concentrations [32] as follows: 2 g/m^2^ pirimiphos methyl in huts 1 and 8, 2 g/m^2^ DDT in huts 3 and 5, and 0.03 g/m^2^ lambda cyhalothrin in huts 7 and 9. By spraying more than one hut with the same compound, and by interspacing sprayed huts with unsprayed ones, we were attempting to also minimise potential differences in mosquito catches between the huts that could bias results. All insecticides were diluted in water and the spraying was performed using standard Hudson X-pert® sprayers (H.D. Manufacturing Company, Chicago, USA) on both the hut walls and ceilings. To avoid contamination, the interception traps and baffles for the IRS huts were fitted two days after spraying, allowing time for the insecticide fumes to settle. Also, all the LLINs used were newly acquired, but were air dried outdoors for twelve hours prior to the start of the experiments so as to prevent any side effects that may be experienced when nets are freshly unpacked.

On the first day of the experiment, the three different LLINs (Olyset®, PermaNet 2.0® or Icon Life®) and the untreated nets were randomly assigned to the nine Ifakara experimental huts, so that each hut regardless of whether it had been sprayed or not, was fitted with either one type of the LLINs or non-insecticidal nets. On subsequent days, the nets were rotated daily to different huts as shown in Table 1, ensuring that at any given time, the different LLINs were either coupled with IRS insecticides in the respective huts, or the nets were used alone in the unsprayed huts. This experimental design also ensured that in the course of these rotations, there were nights when some of the unsprayed huts ended up with just the untreated nets, thereby constituting the experimental controls, against which effects of the other treatments (LLINs alone, IRS alone or LLIN/IRS combinations) could be compared. Two nets of the same type were used per hut, so that each volunteer had his own net each night.

**Table 1:** **A 4-day roster for allocating LLINs and untreated nets in experimental huts sprayed with different IRS insecticides during the spray rounds I and II**^**♣**^

**Spray round I (dry season) treatment rotations**					**Spray round II (wet season) treatment rotations**				
	**Day 1**	**Day 2**	**Day 3**	**Day 4**		**Day 1**	**Day 2**	**Day 3**	**Day 4**
**Hut 1** (DDT)	Olyset^®^	Icon Life^®^	PermaNet^®^	Untreated	**Hut 1** (Pirimiphos methyl)	Olyset^®^	Icon Life^®^	PermaNet^®^	Untreated
**Hut 2** (No IRS)	Untreated	Olyset^®^	Icon Life^®^	PermaNet^®^	**Hut 2** (No IRS)	Untreated	Olyset^®^	Icon Life^®^	PermaNet^®^
**Hut 3** (Lambda cyhalothrin)	PermaNet^®^	Untreated	Olyset^®^	Icon Life^®^	**Hut 3** (DDT)	PermaNet^®^	Untreated	Olyset^®^	Icon Life^®^
**Hut 4** (No IRS)	Icon Life^®^	PermaNet^®^	Untreated	Olyset^®^	**Hut 4** (No IRS)	Icon Life^®^	PermaNet^®^	Untreated	Olyset^®^
**Hut 5** (Pirimiphos methyl)	Olyset^®^	Icon Life^®^	PermaNet^®^	Untreated	**Hut 5** (DDT)	Olyset^®^	Icon Life^®^	PermaNet^®^	Untreated
**Hut 6** (No IRS)	Untreated	Olyset^®^	Icon Life^®^	PermaNet^®^	**Hut 6** (No IRS)	Untreated	Olyset^®^	Icon Life^®^	PermaNet^®^
**Hut 7** (DDT)	PermaNet^®^	Untreated	Olyset^®^	Icon Life^®^	**Hut 7** (Lambda cyhalothrin)	PermaNet^®^	Untreated	Olyset^®^	Icon Life^®^
**Hut 8** (Lambda cyhalothrin)	Icon Life^®^	PermaNet^®^	Untreated	Olyset^®^	**Hut 8** (Pirimiphos methyl)	Icon Life^®^	PermaNet^®^	Untreated	Olyset^®^
**Hut 9** (Pirimiphos methyl)	Olyset^®^	Icon Life^®^	PermaNet^®^	Untreated	**Hut 9** (lambda cyhalothrin)	Olyset^®^	Icon Life^®^	PermaNet^®^	Untreated

^♣^Untreated nets inside unsprayed huts constituted *controls*. Two nets were used per hut, one for each of the two volunteers sleeping in each of the huts.

This design ensured that on a four-day complete block (Table 1), there were three replicates of the experimental controls, three replicates during which the unsprayed huts had each of the three LLIN types on their own (i.e. LLINs alone), two replicates during which the huts had each of the IRS compounds with just the untreated net (i.e. IRS alone) and two replicates during which each IRS compound was combined with each of the LLINs (LLIN/IRS together). The experiments were performed on five consecutive days each week, so that the volunteers and the technicians could rest every Saturday and Sunday of the week. As a result, the rotations were such that the different net types were not always in the same huts on specific days of the week. Though the LLINs were randomly assigned to the different huts initially, their movement between huts each night was predefined, in order to simplify the experiment for the field staff, thus avoiding human error in daily allocation of nets (Table 1). This incomplete-randomisation of treatments is a potential source of bias and was therefore accounted for in the statistical analysis. In readiness for the next evening assignment, the nets were transferred and fitted in the specified huts, immediately after the mosquito collection was completed and huts thoroughly cleaned each morning.

### Mosquito collection

Experiments were conducted from 19.00 hours to 07.00 hours each night. Mosquitoes were collected from the exit traps on eaves and windows and also through indoor resting collections from the inside surfaces and floors of the huts. Mosquitoes found in the exit traps were removed every four hours nightly i.e. at 23.00 hrs, at 03.00 hrs and at 07.00 hrs in the morning. This multiple emptying of the exit traps each night was done to ensure that those mosquitoes which would otherwise have exited the huts did not remain unnecessarily confined in close proximity to the insecticidal treatments, thereby potentially being exposed for a longer period than would occur under natural conditions where mosquitoes would be free to exit local houses and continue foraging.

To ensure that all mosquitoes inside the huts were removed, the morning collection was performed in two steps as follows: first the collectors emptied all the exit traps, collected all mosquitoes resting on the inside hut surfaces and also retrieved any dead mosquitoes found lying on the floors, including under the beds and inside of the bed nets. The collectors then stayed outside the hut for ten minutes, after which they went back inside and repeated the procedure, thus maximising the chance that even those mosquitoes that may have been flying around or missed during the first collection attempt would now be captured. Each indoor collection lasted a minimum of ten minutes on each occasion and continued for as long as the collectors were able to find mosquitoes. In addition to these three main collections (at 2300 hrs, 0300 hrs and 0700 hrs), we also collected mosquitoes that entered and rested within the huts during the day or just before the experiments started, by emptying the traps every evening, at 1800 hrs, before the volunteers went into the huts at 1900 hrs. Since the nets were set up in the specified huts in the morning hours (immediately after the morning collections), those mosquitoes from the evening collections were considered to have been affected by the test interventions in the same way as those mosquitoes entering the huts at night and were therefore added to the nightly totals.

All collected mosquitoes (dead and live) were kept in small netting cages (measuring 15 cm × 15 cm × 15 cm), with 10% glucose solution provided via soaked cotton wool pads. The mosquitoes were kept until the next day inside a holding room located at the same field site where the experimental huts were, at a distance of 80 m from the nearest hut. Mean indoor temperatures inside this holding room were 29.1°C ± 3.0°C during the day and 26.7°C ± 2.3°C at night, while mean relative humidity was 70.6% ± 17.9% during the day and 75.7% ± 13.7% at night. At the end of the holding period, dead and live mosquitoes were segregated. The live ones were then killed with ethyl acetate, after which each group was sorted by taxon and counted.

Malaria vectors, *An. gambiae* s.l and *An. funestus* s.l, together with all other *Anopheles* mosquitoes were first distinguished morphologically from the Culicine mosquito genera, which comprise mainly *Culex quinquefasciatus*, *Mansonia africanus* and *Mansonia uniformis* species [38]. A sub-sample of the *An. gambiae* s.l mosquitoes, was randomly selected for further identification using ribosomal DNA-polymerase chain reaction (PCR) [39] at the Ifakara Health Institute laboratories to distinguish between *An. arabiensis* and *An. gambiae s.s*, the two morphologically indistinguishable sibling species known to occur in the study area [34,35]. Similarly *An. funestus* s.l were examined using PCR to determine sibling species within the group, based on protocols originally described by Koekemoer *et al.,*[40] and Cohuet *et al.*[41].

### Spray rounds

This study was conducted in two spray rounds, the first round being four months long during the dry season of May 2010 to August 2010 and the second being six months long during the wet season of November 2010 to April 2011. To limit the complications of rotating treated and untreated mud panels and ceilings between experimental huts – with potential for cross contamination of treatments, the huts with IRS treatments were fixed for the entire duration of each spray round, and instead only the LLINs were rotated as described above. All the mud panels and *mikeka* ceilings, as well as the inner plastic sheeting usually placed under the sprayed surfaces to ensure that the main framework of the huts are not contaminated [31], were removed for incineration at the end of the first round (dry season tests), and were replaced with fresh material prior the second spray round (the wet season tests), which started three months later.

The experimental procedures in the two spray rounds were generally similar, except for minor incremental improvements in the second round (wet season) relative to the first round (dry season), as follows: 1) in the dry season round both the IRS insecticides and the LLINs were systematically assigned (Table 1), but assignment was fully randomized in the wet season round; 2) the two-step procedure for mosquito collections in the morning was introduced in the wet season tests following observations in the earlier dry season tests, that a small number of mosquitoes were occasionally left behind by the collectors; 3) three of the four windows in all huts were covered during the day, using cut pieces of canvas in the wet season tests to minimise any contamination of exit traps with insecticides from walls or LLINs.

Unlike the specific WHO guidelines [32] regarding the periods after which IRS houses should be re-treated (i.e. 2–3 months for pirimiphos methyl, 3–6 months for lambda cyhalothrin and 6–12 months for DDT), the experiments here measured the efficacy of single treatments over four months in the dry season and over six months in the wet season.

### Protection of participants and ethical approval

Participation of volunteers in the experiments was voluntary, though all participants were compensated for their assistance and time. After full explanation of purpose and requirements of the studies as well as the risks and benefits of participation, written informed consent was obtained from each volunteer prior to the start of all experiments. While inside the experimental huts, the volunteers slept under intact bed nets as a basic protection against mosquito bites. They were also provided with long sleeved, hooded jackets and gumboots, so as to provide additional protection from bites whenever the volunteers stepped outside the nets to collect mosquitoes. In addition, the volunteers were provided with access to weekly diagnosis for malaria parasites and access to treatment with the first-line malaria drug (artemether-lumefantrine) if they had malaria. Perceived adverse effects from exposure to insecticides were monitored by the study co-coordinator and the volunteers were free to leave the study at any time.

Ethical approval for this study was granted by the Institutional Review Board of Ifakara Health Institute (IHRDC/IRB/No.A019), the Tanzania National Institute of Medical Research (NIMR/HQ/R.8aNo1.W710) and the London School of Hygiene and Tropical Medicine Ethical Review Board (Ethics Clearance No. 5552).

### Statistical analysis

#### Analysis of number of mosquitoes entering huts

The nightly total number of mosquitoes of each taxon caught inside the huts or in the exit traps was first calculated by summing live and dead mosquitoes from the respective huts for each collection period, then aggregated to obtain the total catches per night per hut. The totals and median numbers of mosquitoes of each taxon were first compared between huts having the various insecticidal treatments (IRS, LLINs or IRS/LLINs combinations) and the controls (untreated nets in unsprayed huts).

A Poisson log-linear mixed effects model with an individual random effect to account for over-dispersion was fitted to the aggregated data. The fixed effects part of the model was constructed from three factors: treatment (IRS, nets or IRS/net combinations), time (number of months since the start of the experiment), and day order (a variable representing the fact that the net rotations were conducted in a non-randomised sequence on consecutive nights between Mondays and Fridays, but not on Saturdays and Sundays). Random effects were assigned for the variables, hut and day of mosquito collection. Assessment of the fixed effects was done using Wald tests. The relative rates (and 95% confidence intervals) of mosquitoes entering huts with different treatments compared to designated references (i.e. various LLINs alone, various IRS treatments alone, or the experimental controls), were calculated as exponentials of the coefficients generated from the fitted model. Models were fitted using the R statistical software package, version 2.15.0 [42].

#### Analysis of mosquito mortality data

The 24-hour mortality associated with the different insecticidal applications was analysed in two different ways: 1) by considering the proportions of mosquitoes entering individual huts that died on each occasion, a measure suitable for estimating personal household level protection of humans sleeping in the respective treated houses, and 2) by considering the actual numbers of mosquitoes that were killed by the different treatments relative to the controls, a measure also suitable for estimating the likelihood that community level mass protection that can be achieved by these interventions. This is particularly important for interventions that lower vectorial capacity through reducing the survival of vectors, and therefore, lowering malaria incidence among non-users of an intervention in the same community i.e. mass community level effects beyond the households in which they are actually used.

To compare the proportional mosquito mortalities, logistic regression models with random effects were fitted to the data. The fixed effects component of the model was constructed from the following factors: treatment (IRS, nets or IRS/net combinations), time (number of months since the start of the experiment), and day order (to account for the non-randomized daily rotation of nets between huts), and random effects were associated with hut and day. Again tests for fixed effects used Wald statistics. To compare the actual number of mosquitoes killed by the different treatments, the same Poisson random effects log-linear model was used as for the analysis of total mosquito catches. Models were again fitted using the R statistical software package, version 2.15.0 [42].

#### Timing of mosquito exit

This analysis was performed in SPSS version 16 (SPSS inc.) using linear regression of log-transformed mosquito count data. To assess whether the insecticidal treatments affected the times when mosquitoes naturally exited the huts, the mosquito catches in the exit traps at the different periods of the night (i.e. the 1800 Hrs collections, the 1900 Hrs-2300 Hrs collections, the 2300 Hrs-0300 Hrs collections and the 0300 Hrs-0700 hrs collections), were computed as percentages of the total exit trap catches each night, in the different huts. Chi-square analysis was performed to determine if any of the observed percentage increases in early exit were significant relative to the controls (unsprayed huts having non-insecticidal nets).

#### Correlation between total catches and proportional mortality

Finally, to assess whether the huts that had more mosquitoes were also the same huts that had greater proportions dead, i.e. whether the hut design was allowing escape of live mosquitoes and retaining dead or weakened ones, we calculated Pearson correlation coefficients between total catches and percentage mortalities among catches of different species. To accomplish this, simple linear regression analysis was performed on the log transformed *An. arabiensis* catches and proportional mortality computed for these species, in SPSS version 16 (SPSS Inc.) This test was performed only on the *An. arabiensis* mosquitoes.

## Results

### Molecular analysis of mosquitoes

PCR analysis of the *An. gambiae* s.l samples collected during the dry season tests showed that among the 445 successful individual mosquito DNA amplifications, 98.7% were *An. arabiensis* (n = 439) and 1.3% *An. gambiae s.s* (n = 6)*.* All of the 275 *An. funestus* complex mosquitoes collected over the 4 month experimental duration were subjected to molecular analysis, which resulted in 233 successful DNA amplifications. Of these, 96.6% (n = 225) were *An. funestus* Giles, while the remaining 3.43% (n = 8) were *An. rivulorum.* In the wet season collections, PCR analysis was done on 782 *An. gambiae* s.l samples, among which there were 720 successful individual mosquito DNA amplifications. Of these, 95.7% were *An. arabiensis* (n = 689) and 4.3% were *An. gambiae s.s* (n = 31)*.* No molecular identification data was obtained on *An. funestus* during the wet season tests*.* Given the very high proportion of *An. arabiensis* in the study area and the negligible proportion of *An. gambiae s.s.,* all the *An. gambiae s.l.* mosquitoes are hereafter referred to simply as *An. arabiensis*. Besides, since the number of *An. funestus* mosquitoes caught was extremely low throughout the two spray rounds, no further analysis was carried out on this species. The culicines were also not distinguished into species, but recent studies in the area have found that the *Culex* species here mostly consisted of *Cx. pipiens quinquefasciatus* mosquitoes [38] and that the *Mansonia* species consisted mainly of *M. africana* and *M. uniformis*[35]*.*

### Direct protection from mosquito bites (i.e. inhibition of mosquito blood-feeding)

During the dry season tests, less than 0.5% of all live *An. arabiensis* mosquitoes caught in any of the huts (regardless of whether they had LLIN, IRS or untreated nets) and less than 1% of the dead mosquitoes were blood-fed, either fully or partly. Similar results were obtained in the wet season tests, where less than 1% of all live or dead *An. arabiensis* mosquitoes, regardless of the treatments, were blood-fed or partly blood-fed. Therefore, all the IRS treatments (where collectors slept under intact untreated mosquito nets), the LLINs and the controls (which consisted of intact untreated mosquito nets), provided greater than 99% protection from potentially infectious bites by the malaria vector, *An. arabiensis*, for the entire duration of the study (i.e. > 99% feeding inhibition).

### Proportions of mosquitoes caught while exiting the experimental huts relative to proportions caught inside the huts

In both spray rounds, most of the mosquitoes were caught in the exit traps as opposed to inside the experimental huts, regardless of treatment. During the dry season tests, the exit trap catches accounted for at least 94.5% of all mosquitoes collected from any of the huts. The *An. arabiensis* mosquitoes found inside the huts accounted for an average of 5% of the total catches of this species, the maximum percentage indoor catch being merely 6.3%, in the huts having pirimiphos-methyl IRS and untreated nets. Even in the unsprayed experimental huts having only non-insecticidal nets (i.e. the controls), 96.2% of the *An. arabiensis,* 96.9% of *Culex* and 89.5% of *Mansonia* mosquitoes were caught while exiting the huts (i.e. inside the exit traps), as opposed to inside the huts themselves. Similarly, during the wet season tests, collections from the control huts consisted of 98.5% of *An. arabiensis,* 97.8% of *Culex* and 97.8% of *Mansonia* mosquitoes being exit trap catches, meaning that the indoor catches were in all cases less than 5%. Similarly high percentages of mosquitoes caught in treated huts during this round were from exit traps rather than from inside the huts.

### Number of mosquitoes caught in experimental huts with different interventions: analysis of deterrence of mosquitoes from house-entry

Tables 2 and 3 show summary statistics of different mosquito taxa (the malaria vector, *An. arabiensis, Culex* mosquitoes and *Mansonia* mosquito species), that were caught in the experimental huts during the two spray rounds. In the dry season tests, the IRS treatments (DDT, lambda cyhalothrin or pirimiphos-methyl) on their own did not affect the number of mosquitoes entering the huts, though there was a non-significant reduction in *An. arabiensis* catches in huts sprayed with DDT compared to control huts (Relative Rate (RR) and 95% CI = 0.650 (0.351 - 1.202), P = 0.170). Similarly, none of the LLINs on their own reduced malaria vector catches in the huts, except for a non-significant decrease in huts fitted with PermaNet 2.0® nets relative to the controls (RR = 0.731 (0.481 - 1.109), P = 0.140). Regarding addition of IRS onto LLINs, our analysis showed no incremental reduction in mosquito catches in huts having LLINs plus IRS, relative to huts having just the LLINs alone. Where Olyset® or PermaNet 2.0® nets were considered baseline intervention, IRS with DDT or lambda cyhalothrin had no significant effect on *An. arabiensis* catches (P > 0.05), but pirimiphos-methyl doubled the catches relative to Olyset® nets alone (RR = 2.218 (1.194 - 4.118), P = 0.012) or PermaNet 2.0® nets alone (RR = 2.264 (1.218 - 4.204), P = 0.010). However, where Icon Life® nets were used, no IRS insecticide significantly altered malaria vector catches (P > 0.05). Regarding addition of LLINs onto IRS, there was also no incremental reduction in mosquito catches in huts with IRS plus LLINs relative to huts with IRS only. On the contrary, *An. arabiensis* catches increased when Icon Life® or Olyset® nets were added into huts sprayed with pirimiphos-methyl or lambda cyhalothrin (P < 0.05). Icon Life® also increased catches in DDT huts (RR = 1.852 (1.240 - 2.767), P = 0.003), but Olyset® and PermaNet 2.0® nets did not have significant effect (P > 0.05).

**Table 2:** Median numbers (and inter-quartile ranges (IQR)), and the sum of mosquitoes of different taxa caught per night in experimental huts fitted with different IRS and LLIN treatments during the dry season tests

	***Anopheles arabiensis***		***Culex *species**		***Mansonia *species**	
**IRS/LLIN combinations**	**Median (IQR)**	**Sum(n)^**	**Median (IQR)**	**Sum**^**$**^	**Median (IQR)**	**Sum**^**$**^
Untreated nets alone**	66.5 (20.3 - 103.8)	4596 (60)	26.0 (9.0 - 62.3)	2388	9.5 (4.0 - 21.0)	802
Olyset® alone	89.0 (62.3 - 128.5)	6047 (60)	26.5 (10.0 - 65.8)	2701	11.0 (3.3 - 15.8)	743
PermaNet 2.0® alone	67.0 (46.8 - 95.0)	4420 (60)	28.0 (10.3 - 56.3)	2257	7.0 (4.0 - 16.0)	627
Icon Life® alone	79.0 (47.3 - 130.0)	6492 (60)	23.0 (11.3 - 66.8)	2434	13.5 (4.5 - 23.0)	910
Pirimiphos methyl only	89.0 (57.5 -162.8)	4512 (40)	25.5 (10.5 - 51.5)	1437	13.0 (6.3 - 27.3)	669
Pirimiphos methyl and Olyset®	119.5 (71.3 -175.5)	5466 (40)	27.0 (9.8 - 71.3)	1555	10.0 (3.0 - 16.0)	496
Pirimiphos methyl and PermaNet 2.0®	87.5 (60.3 - 139.3)	4691 (40)	22.5 (11.0 - 57.5)	1438	13.0 (7.3 - 23.0)	656
Pirimiphos methyl and Icon Life®	124.5 (78.0 - 216.5)	6022 (40)	33.5 (14.5 - 66.5)	1884	13.5 (7.0 - 30.8)	800
DDT only	45.0 (32.3 -94.3)	2605 (40)	21.5 (10.3 - 48. 3)	1380	10.0 (3.3 - 15.8)	414
DDT and Olyset®	74.5 (45.5 - 102.8)	3162 (40)	26.0 (7.3 - 54.5)	1650	8.0 (3.0 - 15.0)	366
DDT and PermaNet 2.0®	55.5 (38.3 - 74.8)	2728 (40)	24.5 (10.3 - 44.8)	1530	6.5 (3.3 - 17.5)	414
DDT and Icon Life®	94.0 (62.5 - 128.0)	4017 (40)	22.5 (10.0 - 48.5)	1709	10.0 (4.0 - 14.8)	422
Lambda cyhalothrin alone	82.0 (60.8 - 137.8)	4212 (40)	34.0 (9.5 - 67.3)	1673	9.5 (6.0 - 15.0)	533
Lambda cyhalothrin and Olyset®	99.0 (61.0 - 186.8)	5323 (40)	29.0 (7.3 - 51.8)	1355	6.5 (2.0 - 12.5)	361
Lambda cyhalothrin and PermaNet 2.0®	85.5 (41.5 - 141.0)	3931 (40)	31.0 (9.3 - 64.8)	1596	7.0 (3.0 - 17.0)	494
Lambda cyhalothrin and Icon Life®	106.0 (59.3 - 174.5)	5434 (40)	28.5 (7.0 - 56.3)	1477	11.5 (5.0 - 23.0)	598

**^** The term ‘n’ refers to total number of replicates.

^$^ The number of replicates (n) was the same as for *Anopheles arabiensis.*

**Controls refer to unsprayed huts in which volunteers used untreated nets.

**Table 3:** Median numbers (and inter-quartile ranges (IQR)), and the sum of mosquitoes of different taxa caught per night in experimental huts fitted with different IRS and LLIN treatments during the wet season tests

	***Anopheles arabiensis***		***Culex *species**		***Mansonia *species**	
**IRS/LLIN combinations**	**Median (IQR)**	**Sum (n)^**	**Median (IQR)**	**Sum**^**$**^	**Median (IQR)**	**Sum**^**$**^
Untreated nets alone**	64.0 (36.5 - 95.0)	7181 (90)	22.0 (11.0 - 39.5)	2461	5.0 (3.0 - 8.0)	537
Olyset® alone	84.0 (43.0 - 145.8)	9789 (90)	23.0 (10.0 - 39.3)	2498	3.0 (1.0 - 5.3)	380
PermaNet 2.0® alone	61.0 (41.5 - 118.3)	8240 (90)	23.0 (10.0 - 41.5)	2544	3.5 (1.0 - 6.3)	412
Icon Life® alone	105.0 (57.0 - 164.3)	11279 (90)	22.5 (13.8 - 43.3)	2668	6.0 (3.0 - 11.0)	703
Pirimiphos methyl only	85.0 (52.3 - 141.8)	6751 (60)	33.5 (14.5 - 65.8)	3102	9.0 (3.3 - 13.0)	652
Pirimiphos methyl and Olyset®	136.0 (74.8 - 208.3)	9988 (60)	33.5 (16.5 - 74.0)	3384	6.0 (3.0 - 9.8)	437
Pirimiphos methyl and PermaNet 2.0®	94.5 (59.0 - 191.3)	7978 (60)	30.0 (17.0 - 62.3)	3032	7.0 (3.3 - 11.8)	518
Pirimiphos methyl and Icon Life®	144.5 (72.5 - 197.5)	9621 (60)	37.5 (16.3 - 59.5)	3023	9.0 (5.0 - 17.0)	722
DDT only	67.0 (38.3 - 107.8)	4983 (60)	23.0 (12.3 - 46.3)	1828	4.0 (2.0 - 8.0)	365
DDT and Olyset®	76.0 (51.3 - 129.5)	6053 (60)	25.5 (10.3 - 40.8)	1894	3.0 (1.0 - 5.8)	256
DDT and PermaNet 2.0®	72.0 (41.3 - 135.0)	5528 (60)	27.0 (10.3 - 40.5)	1909	4.0 (2.0 - 6.8)	271
DDT and Icon Life®	82.0 (48.5 - 148.5)	6176 (60)	29.0 (15.0 - 43.8)	1925	4.0 (2.3 - 9.0)	438
Lambda cyhalothrin alone	100.5 (51.3 - 178.5)	7535 (60)	20.5 (10.3 - 38.0)	1950	7.5 (4.0 - 13.0)	620
Lambda cyhalothrin and Olyset®	115.5 (65.5 - 207.0)	8947 (60)	23.0 (9.8 - 34.0)	1916	5.0 (2.0 - 9.8)	438
Lambda cyhalothrin and PermaNet 2.0®	100.5 (58.3 - 173.8)	7622 (60)	22.0 (9.5 - 37.8)	2018	6.0 (3.0 - 12.0)	548
Lambda cyhalothrin and Icon Life®	120.0 (71.8 - 243.5)	9784 (60)	23.5 (9.0 - 34.8)	1981	8.0 (5.0 - 15.0)	706

**^** The term ‘n’ refers to total number of replicates.

^$^ The number of replicates (n) was the same as for *Anopheles arabiensis.*

**Controls refer to unsprayed huts in which volunteers used untreated nets.

In the wet season tests, results were generally similar to the dry season (Table 3), and no IRS treatment or LLINs on their own reduced numbers of malaria vectors entering huts, relative to controls. Regarding addition of IRS onto LLINs, there was no reduction in mosquito catches in huts with LLINs plus IRS relative to huts having LLINs alone. Where Olyset® or PermaNet 2.0® nets were the baseline intervention, DDT or lambda cyhalothrin had no effect on vector catches, but pirimiphos-methyl increased the catches relative to Olyset® (RR = 1.744 (1.255 - 2.422), P = 0.001) or PermaNet 2.0® nets alone (RR = 1.542 (1.108 - 2.147), P = 0.010). Again, where Icon Life® nets were used, no IRS significantly reduced or increased *An. arabiensis* catches (P > 0.05). Regarding addition of LLINs onto IRS during the wet season tests, there was also no reduction in mosquito catches in huts with IRS plus LLINs, relative to huts with any of the IRS alone. In huts sprayed with pirimiphos methyl, no net type reduced *An. arabiensis* catches relative to the IRS alone, but the catches increased when Icon Life® (RR = 1.539 (1.199 – 1.976), P < 0.001) or Olyset® nets (RR = 662 (1.305 – 2.117), P < 0.001) were added into these huts. In huts sprayed with DDT or lambda cyhalothrin, adding Olyset® and PermaNet 2.0® nets did not change vector catches, relative to IRS alone (P > 0.05), and in DDT huts, even Icon Life® nets had no statistically significant effect (P = 0.190). However, the catches increased when Icon Life® nets were added to lambda cyhalothrin (RR = 1.317 (1.042 – 1.664), P = 0.021).

The effects of the different treatments on *Culex* and *Mansonia* mosquitoes are also shown in Tables 2 and 3. Generally, there were no significant reductions in *Culex* mosquito catches, except when pirimiphos methyl IRS was combined with Olyset® nets (P = 0.028) or PermaNet® nets (P = 0.010) in the dry season tests, and when lambda cyhalothrin IRS was combined with Olyset® nets (P = 0.043) in the wet season, relative to the controls. We also observed no difference in catches of *Mansonia* mosquitoes between huts with the various treatments relative to the control (P > 0.05) apart from a decrease when using Olyset® nets alone (P = 0.001), or PermaNet® nets alone (P = 0.037) during the wet season tests.

### Proportions of dead mosquitoes caught in experimental huts: analysis of household level protection conferred by the different insecticidal interventions

The proportions of mosquitoes of different taxa (*An. arabiensis*, *Culex* and *Mansonia* species), that died within one day after being collected from experimental huts during the two spray rounds are shown in Tables 4 and 5 and in the Additional file 1. The proportion of dead mosquitoes among the total catches was generally low, the median mortality of *An. arabiensis* for any single treatment or combination being below 30% in either spray round. There was a statistically significant effect of month on vector mortalities caused by all the different treatments (P < 0.05). The relative effects of adding LLINs onto IRS or adding IRS onto LLINs are shown using relative rates (and their 95% confidence intervals) and significance values in Tables 6 and 7.

**Table 4:** Median percentage mortality (and inter-quartile ranges (IQR)), and the sums of mosquitoes of different taxa killed per night in experimental huts fitted with different IRS and LLIN treatments during the first spray round (dry season tests)

	**Mortality of *Anopheles arabiensis***		**Mortality of *Culex *species**		**Mortality of *Mansonia *species**	
**IRS/LLIN combinations**	**Median (IQR)**	**Total dead (n)^**	**Median (IQR)**	**Total dead**^**$**^	**Median (IQR)**	**Total dead**^**$**^
Untreated nets only**	7.1 (3.8 - 14.0)	403 (60)	1.0 (0.0 - 06.2)	77	16.5 (5.5 - 36.9)	170
Olyset® only	11.8 (7.1 - 17.2)	709 (60)	3.9 (0.0 - 08.8)	121	33.3 (6.2 - 50.0)	285
PermaNet 2.0® only	19.5 (13.6 - 26.5)	844 (60)	2.4 (0.0 - 09.0)	87	50.0 (39.6 - 70.1)	343
Icon Life® only	19.0 (12.4 - 27.5)	1028 (60)	2.7 (0.0 - 11.1)	111	50.0 (29.6 - 62.8)	444
Pirimiphos methyl and untreated nets	16.6 (12.1 - 28.7)	836 (40)	9.8 (2.6 - 20.4)	136	42.9 (20.4 - 51.1)	300
Pirimiphos methyl and Olyset®	16.4 (13.1 - 24.9)	980 (40)	7.4 (2.3 - 16.7)	102	41.2 (22.2 - 68.0)	255
Pirimiphos methyl and PermaNet 2.0®	29.0 (18.8 - 36.2)	1196 (40)	6.9 (2.3 - 15.3)	98	71.8 (53.3 - 79.1)	433
Pirimiphos methyl and Icon Life®	21.0 (13.3 - 32.2)	1338 (40)	3.3 (0.3 - 12.5)	108	56.5 (36.6 - 70.3)	433
DDT and untreated nets	14.0 (07.7 - 24.4)	369 (40)	1.4 (0.0 - 13.3)	52	50.0 (18.8 - 66.7)	192
DDT and Olyset®	13.2 (08.8 - 17.2)	411 (40)	3.0 (0.0 - 11.0)	53	46.7 (21.1 - 62.4)	162
DDT and PermaNet 2.0®	17.2 (12.0 - 25.7)	431 (40)	4.2 (0.0 - 12.9)	94	53.8 (36.7 - 66.7)	220
DDT and Icon Life®	12.3 (09.3 - 18.6)	581 (40)	1.8 (0.0 - 08.8)	69	36.1 (20.2 - 50.0)	165
Lambda cyhalothrin and untreated nets	14.8 (10.6 - 22.2)	634 (40)	6.3 (0.3 - 09.9)	106	50.0 (25.0 - 66.9)	304
Lambda cyhalothrin and Olyset®	14.9 (09.6 - 20.6)	755 (40)	6.8 (2.0 - 17.7)	98	66.7 (42.9 - 91.6)	232
Lambda cyhalothrin and PermaNet 2.0®	20.6 (15.3 - 26.5)	802 (40)	6.3 (0.3 - 13.6)	110	64.3 (50.0 - 80.0)	307
Lambda cyhalothrin and Icon Life®	21.6 (16.8 - 26.9)	1055 (40)	5.1 (1.4 - 18.9)	114	62.7 (46.6 - 77.6)	364

**^** The term ‘n’ refers to total number of replicates.

^$^ The number of replicates (n) was the same as for *Anopheles arabiensis.*

**Controls refer to unsprayed huts in which volunteer used untreated nets.

**Table 5:** Median percentage mortality (and inter-quartile ranges (IQR)), and the sums of mosquitoes of different taxa killed per night in experimental huts fitted with different IRS and LLIN treatments during the second spray round (wet season tests)

	**Mortality of *Anopheles arabiensis***		**Mortality of *Culex *species**		**Mortality of *Mansonia *species**	
**IRS/LLIN combinations**	**Median (IQR)**	**Total dead(n)^**	**Median (IQR)**	**Total dead**^**$**^	**Median (IQR)**	**Total dead**^**$**^
Untreated nets only**	10.4 (04.2 - 18.1)	968 (90)	3.3 (0.0 - 10.0)	137	0.0 (0.0 - 27.1)	85
Olyset® only	14.8 (09.3 - 23.9)	1742 (90)	2.9 (0.0 - 10.0)	128	0.0 (0.0 - 42.5)	86
PermaNet 2.0® only	19.7 (11.2 - 30.1)	1644 (90)	3.8 (0.0 - 13.7)	177	26.1 (0.0 - 50.0)	147
Icon Life® only	16.7 (07.2 - 26.4)	2121 (90)	2.3 (0.0 - 11.5)	187	20.0 (0.0 - 46.6)	198
Pirimiphos methyl and untreated nets	23.4 (12.9 - 36.7)	1599 (60)	5.7 (2.5 - 31.8)	272	21.1 (03.9 - 50.0)	119
Pirimiphos methyl and Olyset®	20.3 (12.4 - 31.2)	2171 (60)	7.1 (3.6 - 21.0)	291	31.7 (12.7 - 56.2)	149
Pirimiphos methyl and PermaNet 2.0®	25.0 (14.6 - 36.9)	2146 (60)	9.7 (4.1 - 28.6)	284	50.0 (29.4 - 97.7)	262
Pirimiphos methyl and Icon Life®	21.8 (11.9 - 34.2)	2305 (60)	9.6 (3.8 - 33.6)	316	45.0 (28.6 - 79.5)	282
DDT and untreated nets	17.1 (08.0 - 28.3)	943 (60)	3.6 (0.0 - 14.0)	109	8.0 (0.0 - 38.3)	68
DDT and Olyset®	19.2 (11.6 - 28.1)	1201 (60)	4.3 (0.0 - 11.1)	124	22.5 (0.0 - 50.0)	65
DDT and PermaNet 2.0®	19.4 (12.6 - 34.1)	1171 (60)	4.8 (0.0 - 24.3)	150	33.3 (0.0 - 66.7)	97
DDT and Icon Life®	14.7 (09.7 - 24.1)	1255 (60)	4.6 (0.0 - 10.6)	151	1.5 (0.0 - 30.6)	60
Lambda cyhalothrin and untreated nets	17.8 (10.4 - 28.6)	1431 (60)	9.7 (4.9 - 22.5)	197	21.1 (9.2 - 45.7)	138
Lambda cyhalothrin and Olyset®	14.2 (09.0 - 27.7)	1578 (60)	5.5 (0.0 - 15.4)	157	25.0 (0.0 - 50.0)	136
Lambda cyhalothrin and PermaNet 2.0®	19.0 (10.8 - 33.4)	1768 (60)	7.7 (2.6 - 23.6)	189	50.0 (8.5 - 80.0)	264
Lambda cyhalothrin and Icon Life®	18.4 (09.3 - 26.2)	1893 (60)	8.0 (1.5 - 16.9)	155	33.3 (16.2 - 50.0)	210

**^** The term ‘n’ refers to total number of replicates.

^$^ The number of replicates (n) was the same as for *Anopheles arabiensis.*

**Controls refer to unsprayed huts in which volunteer used untreated nets.

**Table 6:** Relative effects of adding different types of LLINs into huts with different IRS treatments

		**Olyset**® **nets added**		**PermaNet 2.0**® **nets added**		**Icon Life**® **nets added**	
		**RR (95% CI)**	**P value**	**RR (95% CI)**	**P value**	**RR (95% CI)**	**P value**
Unsprayed huts	Dry season	1.315 (1.172 – 1.590)	< 0.001	2.300 (1.981 – 2.672)	< 0.001	2.177 (1.914 – 2.477)	< 0.001
	Wet season	1.328 (1.119 – 1.470)	< 0.001	1.654 (1.575 – 1.736)	< 0.001	1.545 (1.415 – 1.688	< 0.001
Huts sprayed with DDT	Dry season	0.997 (0.769 – 1.129)	= 0.232	1.184 (0.998 – 1.404)	= 0.052	1.221 (0.823 – 1.532)	= 0.079
	Wet season	1.172 (0.972 – 1.323)	= 0.093	1.181 (1.056 – 1.320)	= 0.003	1.227 (0.823 – 1.621)	= 0.081
Huts sprayed with Lambda cyhalothrin	Dry season	0.841 (0.739–0.956)	= 0.008	1.461 (1.285 – 1.662)	< 0.001	1.239 (1.094 – 1.405)	< 0.001
	Wet season	1.157 (0.921 – 1.454)	= 0.210	1.698 (1.354 – 2.129)	< 0.001	1.433 (1.190 – 1.725)	< 0.001
Huts sprayed with Pirimiphos methyl	Dry season	0.994 (0.866–1.141)	= 0.930	1.773 (1.544 – 2.036)	< 0.001	1.387 (1.224 – 1.571)	< 0.001
	Wet season	1.321 (1.126 – 1.549)	= 0.001	1.262 (1.075 – 1.481)	= 0.004	1.386 (1.182 – 1.626)	< 0.001

The table shows relative rates (RR) and associated 95% confidence intervals of increased mortality of *Anopheles arabiensis* in huts with IRS plus LLINs, compared to huts with IRS supplemented only with untreated nets.

**Table 7:** Relative effects of adding different IRS treatments in huts with different LLIN types

		**Adding IRS with pirimiphos methyl**		**Adding IRS with lambda cyhalothrin**		**Adding IRS with DDT**	
		**RR (95% CI)**	**P value**	**RR (95% CI)**	**P value**	**RR (95% CI)**	**P value**
Huts with Untreated nets	Dry season	2.200 (1.734 – 2.792)	< 0.001	1.920 (1.582 – 2.330)	< 0.001	1.730 (1.398 – 2.140)	< 0.001
	Wet season	2.208 (1.821 – 2.677)	< 0.001	1.551 (1.274 – 1.887)	< 0.001	1.444 (1.181 – 1.766)	< 0.001
Huts with Olyset® nets	Dry season	2.218 (1.194 – 4.118)	= 0.012	1.717 (0.965 – 3.055)	= 0.538	0.940 (0.510 – 1.732)	= 0.843
	Wet season	1.375 (1.143 – 1.654)	= 0.001	1.075 (0.889 – 1.301)	= 0.455	1.174 (0.968 – 1.425)	= 0.103
Huts with PermaNet 2.0® nets	Dry season	2.264 (1.218 – 4.207)	= 0.010	1.313 (0.734 – 2.349)	= 0.359	0.920 (0.524 – 1.797)	= 0.924
	Wet season	1.420 (1.179 – 1.710)	< 0.001	1.173 (0.969 – 1.419)	= 0.103	1.031 (0.849 – 1.253)	= 0.756
Huts with Icon Life® nets	Dry season	1.401 (1.169 – 1.680)	< 0.001	1.093 (0.922 – 1.295)	= 0.306	0.767 (0.636 – 0.925)	= 0.006
	Wet season	1.237 (1.029 – 1.486)	= 0.023	1.008 (0.835 – 1.217)	= 0.972	0.995 (0.821 – 1.205)	= 0.958

The table shows relative rates (RR) and associated 95% confidence intervals of increased mortality of *Anopheles arabiensis* in huts with LLINs plus IRS, compared to huts with nets alone.

**Figure 1:**
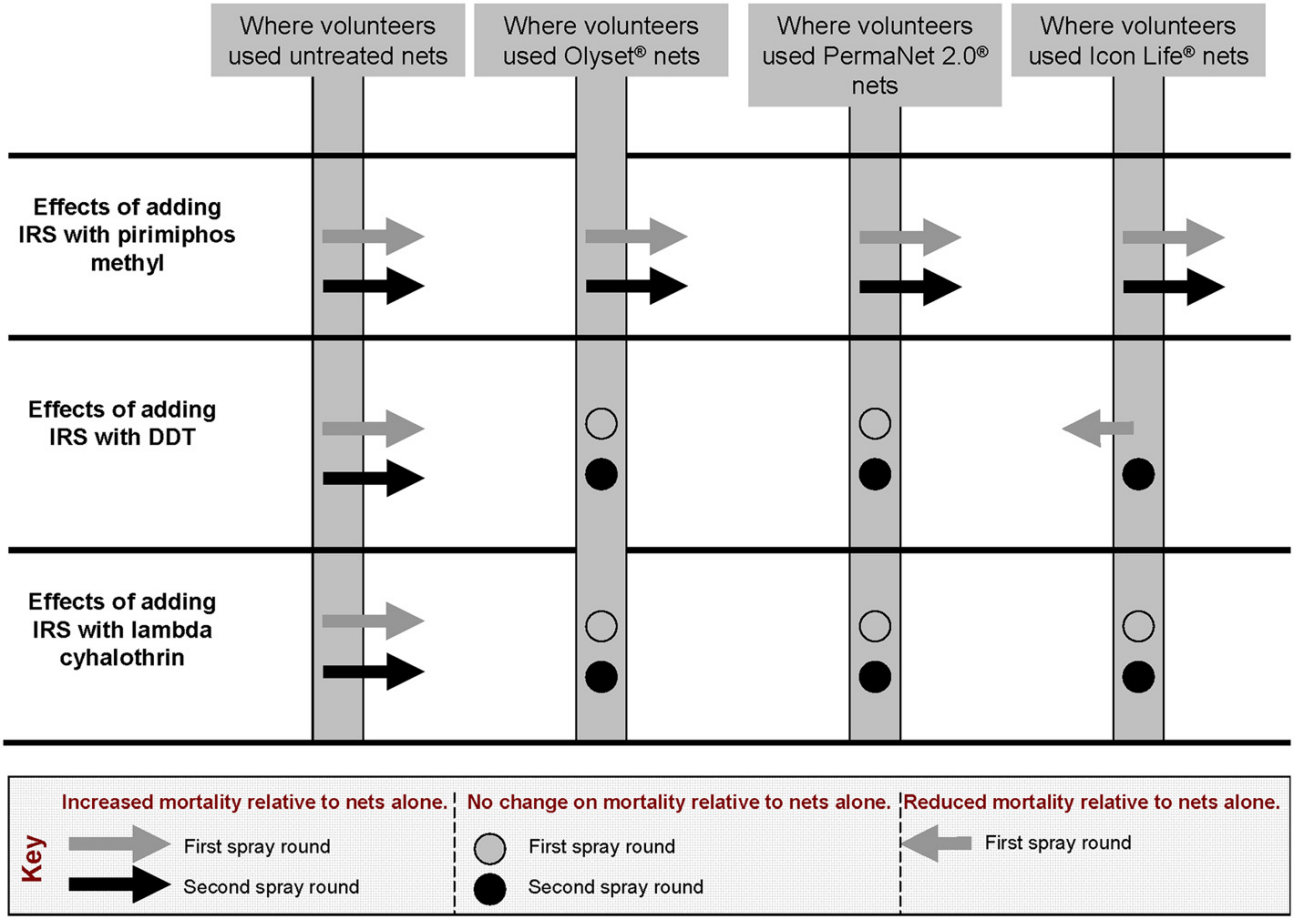
**Summary of the observed changes on proportional mortality of the malaria vector, *Anopheles arabiensis, *when different IRS insecticides are introduced in situations where volunteers were already using different net types.** Summaries are shown for both dry season (first spray round) and wet season (second spray round) tests.

In the dry season tests, all IRS treatments, all LLINs and their combinations significantly increased the proportion of dead *An. arabiensis* relative to controls. The most toxic IRS relative to controls was pirimiphos methyl, followed by lambda cyhalothrin, then DDT. Of the LLINs, PermaNet 2.0® were the most toxic relative to controls, followed by Icon Life®, then Olyset® nets. Regarding addition of IRS onto LLINs (Figure 1 and Table 7), data from the dry season tests showed that there was mostly no statistically significant increase of proportional mortality among malaria vectors caught in huts having LLINs plus IRS, relative to huts having LLINs alone, except where IRS treatment was pirimiphos-methyl. Regarding addition of LLINs onto IRS (Table 6), we observed in the dry season tests that unlike in cases of adding IRS onto LLINs, there were mostly statistically significant increases in proportional mortality among malaria vectors caught in huts with IRS plus LLINs relative to mortality in huts with IRS alone, except where the specific LLINs added were Olyset®, which had no such effects, and where the IRS was done using DDT, which also seemed to counteract additional mortality when used in combination.

In the wet season tests, results were generally similar to results from the dry season, with regard to proportional mortality of malaria vectors. All IRS treatments, LLINs and LLIN-IRS combinations significantly increased the proportion of dead *An. arabiensis* mosquitoes, relative to the controls. Like in the dry season tests, the most toxic IRS, relative to the control was pirimiphos methyl (Tables 6 and 7). Besides, the most toxic LLINs relative to controls were again PermaNet 2.0®, followed by Icon Life®, then Olyset® nets. Regarding addition of IRS onto LLINs (Figure 1 and Table 7), analysis of the wet season data revealed that in most cases, there was no increase of malaria vector mortality in huts having LLINs plus IRS, relative to huts having LLINs alone, except where the specific IRS treatment was pirimiphos-methyl. Regarding addition of LLINs onto IRS (Table 6), we observed again in the wet season tests that proportional mortality of malaria vectors was significantly higher in huts having IRS plus LLINs, relative to huts having just IRS alone, except in certain cases where the LLINs added were Olyset® nets and where the IRS was based on DDT.

Although toxicity of all treatments to *Culex* mosquitoes was evidently lower than toxicity to *An. arabiensis*, data from both rounds show that relative to controls, higher proportions of *Culex* were killed in huts sprayed with pirimiphos methyl or lambda cyhalothrin (P ≤0.003) and in huts having DDT coupled with PermaNet 2.0® nets (P < 0.001). Also, higher proportions of *Mansonia* mosquitoes died in huts with DDT coupled with PermaNet 2.0® in the dry season round (P = 0.001). Similarly in the wet season round, all treatments except DDT alone (P = 0.245) or DDT coupled with Icon Life® nets (P = 0.374) killed significantly higher proportions of *Mansonia* mosquitoes than controls.

### Actual number of mosquitoes killed per night in different huts: analysis of communal protection conferred by the different insecticidal interventions

Our field data had shown that huts with certain insecticidal treatments, notably pirimiphos methyl had consistently higher densities of malaria mosquitoes than the control huts (Tables 2 and 3). It was likely that such huts would constitute more effective ‘lure and kill’ stations for host seeking vectors, by letting in large numbers of the vectors and killing significantly proportions of these. Therefore, in addition to computing the proportional mortality among mosquitoes that entered different experimental huts, we also analysed and compared the actual numbers of mosquitoes that were killed per hut per night, regardless of the total numbers of mosquitoes actually entering the huts. Unlike proportional mortality which is useful for comparing between products and estimating household level protection conferred to users, the actual total number of mosquitoes killed by any intervention can indicate the extent of mass communal protection achievable from that intervention; since by killing potentially infectious vectors, vectorial capacity is reduced within that community. More detailed information on the absolute numbers of *An. arabiensis, Culex* and *Mansonia* mosquitoes killed by different interventions is provided in Tables 4 and 5, and in the Additional file 1.

In the dry season tests, all the treatments and their combinations increased actual numbers of *An. arabiensis* mosquitoes killed per hut per night, relative to controls except DDT when used alone (P = 0.311) or in combination with Olyset® nets (P = 0.063). Regarding addition of IRS onto LLINs, there were no increases in numbers of malaria vectors killed nightly in huts having LLINs plus IRS, relative to huts with LLINs alone, except where the IRS treatment was pirimiphos-methyl.

Regarding addition of LLINs onto IRS, we observed that unlike in the case of adding IRS onto LLINs, but similar to findings on proportional mortality, there were mostly significant increases in numbers of malaria vectors killed nightly in huts with IRS plus LLINs, relative to huts with just IRS, except in a few cases where the LLINs were Olyset® or where IRS treatment was DDT (Additional file 1). In the wet season tests, findings showed that regarding actual numbers of malaria vectors killed per hut per night, were also similar to those obtained in the dry season. All IRS treatments, all LLINs and their combinations significantly increased numbers of dead *An. arabiensis*, relative to controls. When IRS was added to LLINs, data from the wet season tests showed that IRS with pirimiphos methyl or lambda cyhalothrin (in some cases), but not DDT, significantly increased numbers of malaria vectors dying per night in huts with any LLIN. Regarding addition of LLINs onto IRS treatments, the total number of malaria vectors killed per night was mostly higher in huts having IRS plus LLINs, compared to huts with IRS alone, except in certain specific cases where the LLINs were Olyset® nets or where the IRS was based on DDT.

### Time of the night when mosquitoes exit huts: analysis of irritant effects of the different insecticidal interventions

**Figure 2:**
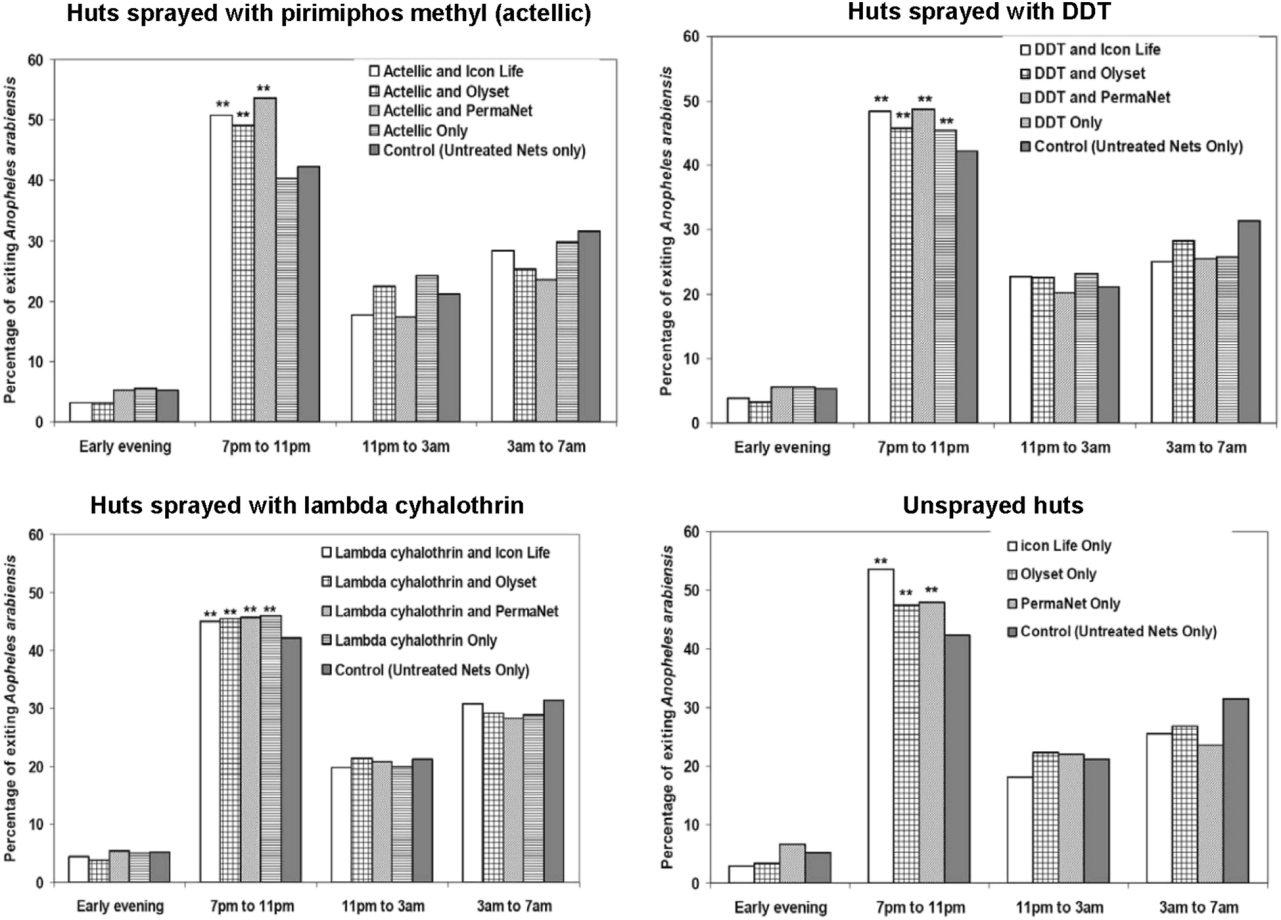
**Effects of IRS/LLIN applications on the time when *Anopheles arabiensis *exited volunteer-occupied experimental huts during the dry season tests.** Bars marked with two stars (**) denote irritant applications that caused significantly more mosquitoes (P < 0.05) to exit earlier than in the controls.

**Figure 3:**
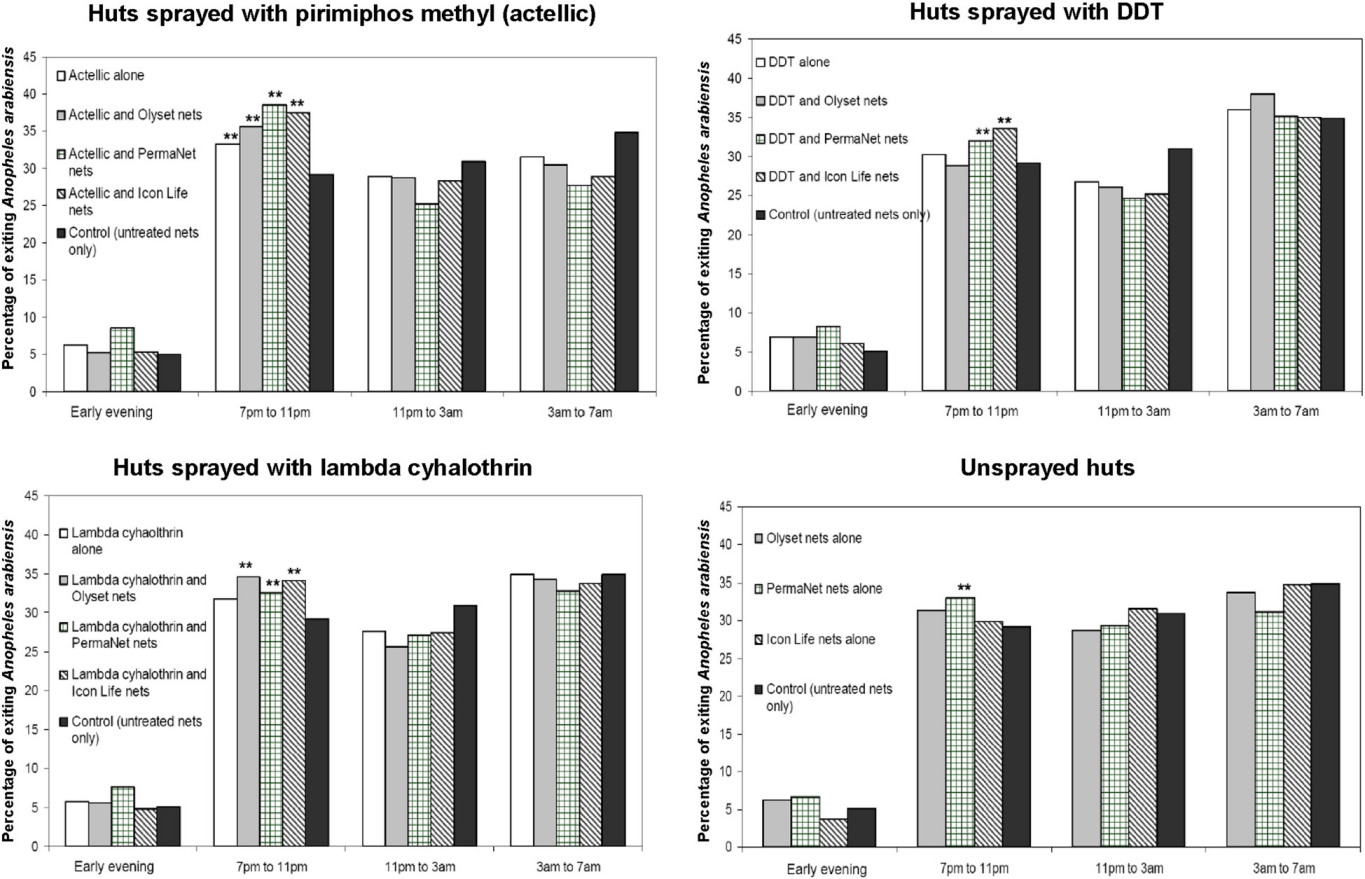
**Effects of IRS/LLIN applications on the time when *Anopheles arabiensis *exited volunteer-occupied experimental huts during the wet season tests.** Bars marked with two stars (**) denote irritant applications that caused significantly more mosquitoes (P < 0.05) to exit earlier than in the controls.

Considering only those mosquitoes that were caught exiting, the tendency of malaria vectors to exit human-occupied experimental huts earlier than normal was examined in experimental huts with the different insecticidal treatments, relative to the controls. Figure 2 and Figure 3 show these patterns of mosquito exit, during the first and the wet season tests respectively.

During the first spray round (dry season), most of the mosquitoes caught exiting the control huts consisted of those caught between 7 pm and 11 pm. However, this pattern shifted slightly but significantly in the huts with any of the LLINs or IRS, such that the proportion exiting between 7 pm-11 pm was now significantly increased (P < 0.05). The only exception was with pirimiphos methyl IRS used alone, which did not have this irritant effect (Wald *χ*^2^ = 1.549, P = 0.213, DF = 1). The general exit pattern, however, remained unchanged, meaning that most of the mosquitoes were still mostly exiting during the same time period as if there were no insecticidal applications within the huts (Figure 2). There was also apparent but marginal increases in early exit when the IRS and LLINs were used together, relative to whenever either the LLINs or the IRS were used alone (Figures 2 and 3).

The greatest shift towards early exit was observed in huts having both pirimiphos methyl IRS and PermaNet 2.0® nets (Wald *χ*^2^ = 65.095, P < 0.001, DF = 1), and in huts having Icon Life® nets alone (Wald *χ*^2^ = 65.322, P < 0.001, DF = 1), both of which resulted in 53.6% of the *An. arabiensis* mosquitoes exiting between 7 pm and 11 pm compared to the controls where an average of 42.9% were exiting at the same period. Many of the other treatments, including DDT, caused less than 10% increase in this early exit rate.

In the wet season tests, a higher proportion of the *An. arabiensis* exited from the control huts at dawn, between 3 am and 7 am, perhaps due to climatic difference between the two rounds. As shown in Figure 3, the greatest percentage of the exiting mosquitoes was observed to be between 3 am and 7 am. However, some of the LLINs, IRS or their combinations shifted this pattern so that most of the mosquitoes were now exiting earlier during the night, i.e. between 7 pm and 11 pm (Figure 3). Overall, there was, just like in the dry season tests, an apparent but marginal increases in early exit when the IRS and LLINs were used together, relative to whenever either the LLINs or the IRS were used alone (Figures 2 and 3). PermaNet 2.0® nets used alone induced this excessive early exit by a significant margin relative to the controls (Wald *χ*^2^ = 7.263, P < 0.007, DF = 1). Of the different IRS treatments, only pirimiphos methyl induced excess early exit by a significant margin relative to controls (Wald *χ*^2^ = 8.56, P < 0.003, DF = 1). All the IRS/LLIN combinations also increased early exit relative to the controls, with the exception of DDT plus Olyset® nets (Wald *χ*^2^ = 0.044, P = 0.834, DF = 1). Similar to the dry season tests, the greatest shift towards early exit in this round was also observed in huts having pirimiphos methyl IRS combined with PermaNet 2.0® nets (Wald *χ*^2^ = 44.329, P < 0.001, DF = 1), which resulted in 38% mosquitoes exiting in the period between 7 pm and 11 pm, compared to 29% exiting controls at the same period.

### Relationship between total number of mosquitoes caught and the proportions that died

To assess whether the huts that had more mosquitoes were also the huts that had greater proportions of dead mosquitoes, which would suggest that live mosquitoes might be escaping or not being captured, and that mainly the dead or weakened mosquitoes were remaining – indicating a design flaw in the huts, the statistical correlation between the total malaria vector catches and proportional mortality among these catches was examined. If the high mosquito catches in treated huts were due to the fact that live mosquitoes were leaving the huts, and that mainly the dead or weakened mosquitoes were remaining, then one would have expected there to be a significant relationship between the two variables, total catch and proportional mortality. However, it was observed that no statistical associations existed between these variables (P > 0.05) except for a weak association in huts with pirimiphos methyl coupled with Olyset® nets that was statistically significant (R^2^ = 0.08, P = 0.027).

## Discussion

All the accrued achievements in malaria control notwithstanding, many experts now believe that sustainable control of the disease in many parts of Africa will require not only the introduction of new complementary strategies, but also optimization of existing methods, particularly LLINs and IRS [25,43-47]. Combining LLINs and IRS in same households is already widely practiced in many highly endemic areas, usually with the intention of achieving greater health benefits, even though it is much more costly to implement both interventions, than to use either LLINs or IRS, alone. Unlike the previous studies on combining insecticidal interventions [23,27,28], this is the first that directly compares multiple combinations of LLINs and IRS at household level. Moreover, the study was conducted in an area dominated by *An. arabiensis* (with a high degree of physiological susceptibility to common insecticides), which is increasingly considered a major driver of residual malaria transmission in many localities [33,46,48,49].

To answer the key question of whether LLIN-IRS combinations can have greater effects on malaria exposure, than either LLINs alone, IRS alone or untreated nets alone, we considered two different but complementary approaches. First, we considered situations where IRS is already in place as the baseline intervention and then examined possible benefits of adding different LLINs or untreated nets. Second, we considered situations where LLINs (or the untreated nets) are already being used, and then assessed possible additional value of IRS with different chemicals. The methodology used for the study readily enabled consideration of either of these two perspectives, in the same study. Obviously, given the widespread use of LLINs in Africa today [8], it makes more practical sense to examine the value of IRS when added onto LLINs rather than LLINs when added onto IRS.

In the first scenario, where IRS is already in place, this study shows that addition of LLINs would be clearly beneficial by enhancing direct personal protection against bites (as we found that > 99% of collected mosquitoes were unfed) and to a small extent by killing additional malaria mosquitoes in the houses. Preventing mosquitoes from entering houses (i.e. mosquito deterrence) on the other hand is not an important protective property of LLINs, as we observed no significant reduction in malaria vector catches as a result of adding any of the LLINs onto any of the different IRS treatments. As depicted by the generally low mortality rates of malaria vectors in this study, both intact LLINs and untreated nets, when used correctly and consistently appear to function primarily by preventing mosquitoes from feeding upon hut occupants. Even when added onto IRS, any additional benefits of LLINs in similar epidemiological scenarios would be primarily due to this direct prevention of mosquito-human contact, and only marginally due to the insecticidal toxicity of the LLINs to the mosquitoes. The limited efficacy of LLINs against *An. arabiensis* has previously been reported in Tanzania [50], and may suggest that these otherwise effective tools may be subject to undesirable limitations in residual transmission settings dominated by this vector species. Moreover, in situations where LLINs are added onto IRS, the physical barrier effect of the nets (i.e. prevention of human-mosquito contact) could also reduce performance of the IRS, as unfed mosquitoes are less likely to rest on the treated surfaces inside the dwellings for long enough to guarantee being killed by the sprayed chemicals.

In the second scenario, where LLINs are already being used, which (given the widespread use of LLINs) is the more common situation in Africa today, rather than vice versa [8], the value of LLIN/IRS combinations is much lower than in the first scenario. This study shows that additional protection from IRS treatments, if any, would primarily be the result of modest increases in toxicity, resulting in slightly more mosquitoes being killed relative to situations where only the LLINs are used (Figure 1 and Table 7). Of the tested IRS compounds, only pirimiphos methyl consistently increased the proportional mortality by a significant margin relative to what is achievable with LLINs used alone. Lambda cyhalothrin or DDT did not have any such effect relative to any of the LLINs used alone. In fact, in one interesting scenario, where DDT was added onto Icon Life® nets, overall proportional mortality of *An. arabiensis* in the experimental huts was actually less than in huts with just the Icon Life® nets alone (Figure 1), probably due to increased irritancy reducing the amount of time vectors spent in contact with nets. It seems, therefore, that where people already use any of the LLINs, additional improvements by IRS can be obtained only where the chemical of choice is either pirimiphos methyl (as used in this study), or some other approved active ingredient with similarly high or higher toxicity, and preferably low irritancy to the mosquitoes.

This suggestion actually matches current proposals by the WHO and many experts, who are concerned that overexposing mosquitoes to insecticides of the same class, would accelerate insecticide resistance and antagonise efforts to preserve efficacy of LLINs and IRS [19,23,30]. Since pirimiphos methyl is an organophosphate, combining it with any LLINs (all of which are currently pyrethroid-based), would therefore not only provide additional household protection, but it would also potentially mitigate against the rise and spread of resistance alleles among vector populations [19,30]. Indeed, recent hut trials in Benin provided some indirect evidence for this argument, as combinations of LLINs with chlorfenapyr, a pyrole insecticide, were shown to kill greater proportions of mosquitoes bearing insecticide resistance genes, relative to LLINs used on their own [23]. Resistance management is therefore one of the reasons that LLIN-IRS combinations may still be necessary in even in settings with some settings.

With regards to reducing mosquito entry into huts, we observed that whereas IRS using DDT, or PermaNet 2.0® nets would provide minor additional household level protection by deterring mosquitoes from entering huts (as observed during the dry season tests), this effect was not statistically significant, and was also not a property of any other IRS chemical or LLINs tested here. The reason for this low observed deterrence may be the design of the experimental huts that we used here, which had steel and iron sheet overhangs above the eave spaces [31], unlike previous huts which had thatched roof overhangs [51]. Evidence suggests that DDT particles blown onto thatch overhangs [51], or treatment of these overhangs [52] can prevent hut entry by mosquitoes. In our studies however, the huts were not fitted with thatch panels as it was expected that dust would build up on the netting baffles and create a similar deterrent effect. It is also interesting to observe that deterrence from DDT was observed only in the first spray round (dry season), but not in the wet season round during which the practice of closing off window traps at day time was being implemented. This practice could have reduced air movement through the houses and minimized the occurrence of deterrent effects.

It has previously been suggested that household level protection can be enhanced if highly deterrent IRS, which minimise mosquito entry into the houses, are coupled with highly toxic LLINs, which kill most of the mosquitoes that manage to enter the houses and attack net users inside [19]. In such cases, high intervention coverage could ensure that mosquitoes deterred from protected households do not find feeding opportunities in unprotected households [19]. However, a closer examination of available evidence suggests that successful IRS applications [9,53,54] and LLIN campaigns [33,48] which have led to elimination or near elimination of specific indoor feeding and resting vectors, did so mostly because they let in and killed large proportions of these vectors consistently over a period of time. Therefore, for long-term communal benefits, purely toxic insecticidal interventions are considerably better than insecticidal interventions that are also partly deterrent to the vectors [55], if the vectors feed and rest indoors and therefore make adequate contact with the interventions. In this current study, we observed that DDT, the only IRS with potential to deter *An. arabiensis* from entering huts, would actually also reduce overall proportional mortality (Figure 1) of these vectors in huts with specific LLINs (in this case Icon Life® nets). This observation, in addition to the likelihood of cross-resistance developing between DDT and pyrethroids when used together [56], reinforces the disadvantage of using DDT for IRS in households where pyrethroid based LLINs are already being used.

Another possible source of additional protection could be the irritant effects of certain IRS chemicals. This study shows that compared to situations where volunteers used untreated nets, there was an enhanced rate of early exit by malaria vectors from treated huts, though the percentage increase was marginal (Figures 2 and 3). Nevertheless, significant levels of early exit were also being observed in the control huts. It appears therefore that this behaviour was only minimally associated with insecticide induced irritancy. Instead, the main reason mosquitoes were not spending long periods inside different experimental huts was that either they had failed to feed and left the huts to continue foraging (since we also observed > 99% feeding inhibition in all huts), or because it was the natural behaviour (exophily) of the local *An. arabiensis* populations in the study area.

As already mentioned above, the percentage vector mortalities in this study were lower than in many previous studies, the results of which were presented in the supplementary online materials accompanying the review article by Okumu and Moore [19]. Concurrent insecticide susceptibility tests showed that the local vector populations were mainly susceptible, albeit with slightly reduced susceptibility to pyrethroids; the *An. arabiensis* here were 100% susceptible to DDT, 95.8% susceptible to deltamethrin, 90.2% susceptible to lambda cyhalothrin and 95.2% susceptible to permethrin [36]. Moreover, results of cone bioassays conducted on the hut surfaces immediately after the spraying also showed 90% mortality among *An. arabiensis* exposed to walls treated with lambda cyhalothrin, 97.5% on DDT sprayed walls and 100% on pirimiphos methyl sprayed walls, suggesting satisfactory quality of spraying and susceptibility of the mosquitoes, which had been newly colonised from the same study area [36]. We therefore do not consider insecticide tolerance or resistance, as an explanation for the low vector mortalities observed in this study.

Instead, we can infer, based on the study design and the observed mosquito exit times (Figure 2 and Figure 3), that the low vector mortalities (which have also been reported in some recent studies on LLINs [50]) may be related to the behaviour of *An. arabiensis,* which readily exited the huts on realization that the intended blood hosts were covered by nets and therefore unavailable. In this study are linked to the fact that most of the collected mosquitoes (> 95% in all cases) were actually caught while exiting the huts. The egress was mostly occurring soon after the mosquitoes entered the experimental huts and was observed in both treated and control huts (Figures 2 and 3). This observation coupled with the fact that the collections were done multiple times a night (i.e. every four hours), suggests that the mosquitoes visited the huts normally, but exited soon afterwards, most likely because they had not been successful in finding any blood meals in the huts, as suggested above. Clearly, the mosquitoes were not spending sufficient time in the huts to receive a fatal exposure to the insecticides. While it is natural that unfed mosquitoes may continue their host seeking activity elsewhere [57], what is obvious is the indication that these mosquitoes, or at least the local *An. arabiensis* populations, tended to give up on any hosts found to be protected with nets, and therefore readily flew out of the huts. This observation matches the known behaviour of *An. arabiensis,* which can be fairly opportunistic [58], and could therefore also explain why *An. arabiensis* populations are affected to a lesser extent by insecticidal nets, than *An. gambiae* s.s. [33,50].

The other two possible and related explanations for the low mortalities of malaria vectors as observed in this study are: 1) the fact that we used a different experimental hut design, which is large and is fitted with baffles on eaves, so that they more representatively mimic local houses and the vector behaviour around them [31], and 2) the fact that all the nets used in this study were intact (un-holed) nets; such that even the control huts had intact untreated nets rather than no nets at all. This means mosquitoes were restricted from feeding upon the hut occupants, and were more likely to exit the huts and continue host seeking. Indeed, less that 1% of the collected mosquitoes were either fully or partly blood fed, which could have translated to very few mosquitoes resting in these huts to digest their blood meals, a situation which would possibly have guaranteed longer contact with the treatments and caused higher post-feeding mortality as shown in some previous IRS studies [23,51]. On one hand therefore, this experimental set-up, with intact nets as controls may, to a certain degree, misrepresent real life situations where poor care of LLINs can lead to damage after just a few months of use, and could therefore have resulted in an underestimation of toxicity. Nevertheless, it underlines the importance of sufficiently frequent bed net replacement programs for malaria control, coupled with health education on appropriate net use practices, as the intact nets were remarkably protective in this study. Perhaps most importantly, the results indicate that in situations where a great proportion of residual malaria transmission is driven by *An. arabiensis,* LLINs and IRS will have limited insecticidal effects, over the physical protection from mosquito bites offered by the nets when intact.

In addition to computing the proportional mortality among mosquitoes that entered different experimental huts, we also examined and directly compared the actual numbers of mosquitoes killed in huts that had the different treatments, relative to the controls. The main reason for this was to obtain an idea of what the contributions of these insecticidal applications would be in terms of mass community level benefits; since such mosquitocidal interventions can remove large numbers of vectors from the malaria transmission cycle and reduce the probability that vectors live long enough to become infective [59-62]. This study has shown that LLIN/IRS combinations based on pirimiphos methyl as the IRS would result in the greatest community level effect, but that significant benefits are also achievable in huts with lambda cyhalothrin when supplemented with either Icon Life® or PermaNet 2.0® nets. Highly effective contact toxicants such as IRS with pirimiphos methyl or intact PermaNet 2.0® and Icon Life® nets, or combinations thereof, which let in and kill large numbers of malaria mosquitoes, would provide a greater community level impacts than interventions that let in and kill fewer mosquitoes due to deterrent or irritant modes of action. This point of view has been expressed by malariologists for many years, including Curtis and Mnzava [53], who suggested over a decade ago that non-irritant insecticides should be favoured for IRS over the pyrethroids because the latter make insects leave the site of treatment, thus reducing mosquito mortalities.

Perhaps the most important reason that people use nets is to prevent mosquito bites. For most users, this generally includes nuisance mosquitoes such as many of the *Culex* and *Mansonia* mosquito species, which can also transmit a number of neglected tropical infections [38]. Based on the results of this study, all nets if used consistently and are kept intact, can prevent mosquito feeding by more than 99% at household level, matching previous demonstrations of considerable efficacy of untreated nets [33,63,64]. It is therefore appropriate to emphasize that in situations where IRS is already being used, intact nets, even if untreated, can significantly improve protection relative to the IRS alone, even though no added advantage should be expected from adding IRS where most people already correctly use LLINs or untreated nets that are intact, unless the IRS is highly toxic. Future malaria control strategies could therefore be enhanced by increasing the focus on: 1) tougher and longer lasting net fibres, 2) regular net replacement programs, 3) ensuring that nets are made available in appropriate sizes to adequately cover sleeping spaces and 4) complementary behaviour change programs to ensure proper and consistent use of nets.

This study adds to the evidence [19,20,36] that addition of IRS may not always be advantageous in situations where large proportions of people already correctly use LLINs. It also suggests that even where IRS is to be introduced to complement LLINs, careful selection of the active ingredient is necessary. The selected IRS insecticide should be that which functions primarily as a contact toxicant and can kill large proportions of mosquitoes that enter huts, rather than chemicals that also deter or irritate the mosquitoes. Though this study suggests that pirimiphos methyl was the only one of the tested IRS candidates which would provide greater value than LLINs alone, any other non-pyrethroid IRS insecticide with similar properties could potentially offer similar benefits. Whereas this was merely a household level study conducted in experimental huts, mathematical simulations parameterised using these same datasets [65], also suggested similarly limited additional value of adding IRS with pyrethroids or DDT, in communities representative of current African malaria scenario, where most households already have nets, and the endophillic *An. gambiae s.s.* populations are on the decline [33,66-68]. Moreover, there have not been many epidemiological studies attempting to answer this question, but a recent randomised controlled trial in Benin has also shown limited additional benefit of adding IRS with bendiocarb, or carbamate treated wall sheeting onto LLINs [28].

An important aspect of this current study is that, having tested several IRS compounds, it has been possible to determine that while IRS with DDT or pyrethroids are not likely to confer additional value, IRS with highly toxic organophosphates can still be useful albeit modestly. Therefore, the results should not be interpreted to mean that adding IRS onto LLINs is always redundant. On the contrary, careful selection of candidate IRS insecticide should be emphasised, and IRS should be considered as a secondary measure, after LLINs have been provided. Moreover, given that IRS chemicals decay rapidly from sprayed surfaces and are recommended for re-treatment within just a few months [32], carefully timed, properly targeted [69] and well managed re-spray campaigns, could maximise the value of IRS, especially when responding to malaria epidemics or vector population rebounds, and more so if such epidemics or rebounds are mediated by the more endophilic and anthropophilic vectors such as *An. gambiae s.s.* and *An. funestus*.

## Conclusion

Where the primary malaria vector is *An. arabiensis,* there are minimal additional protective benefits to be gained from adding IRS with DDT or lambda cyhalothrin into houses where people properly use any of the tested LLINs (Olyset® nets, PermaNet 2.0® nets or Icon Life® nets). Given the available range of insecticides for malaria control, combining pyrethroid based LLINs with IRS would be effective only if the IRS of choice is highly toxic to the malaria vectors and preferably non irritant, but even in such situations, the additional protection would be modest, requiring that any decision to combine or not combine LLINs and IRS is made in consideration of the cost of these marginal benefits. Of the tested IRS chemicals the only one that would provide any form of enhanced protection relative to LLINs alone is the organophosphate, pirimiphos methyl. The combination of this organophosphate with any of the current LLINs (all of which are currently pyrethroid-based) might also be suitable as an insecticide resistance management strategy. In contrast, addition of LLINs always enhances personal protection, by preventing mosquito bites (i.e. feeding inhibition) and by slightly increasing mosquito mortality in the houses. Even intact untreated nets, by merely preventing mosquito bites, can constitute an effective complementary intervention to be used alongside IRS in communities where the main vector is *An. arabiensis*, though such a strategy would have limited application in mitigating insecticide resistance. We therefore recommend that where resources are limited, the focus of malaria vector control should be to ensure that everyone in a malaria risk area uses an LLIN properly and consistently. This will require parallel community education programs, as well as a regular net replacement strategy to ensure that the nets remain intact. These findings should, however, not be overly generalised, as there are still some scenarios where combined insecticidal interventions would be key. For example, where it is not possible to provide everyone with LLINs, where the LLINs cannot be maintained in an intact state or regularly replaced, and in epidemic prone situations where transmission is driven by endophilic and anthropophilic vectors such as *An. gambiae s.s.* and *An. funestus*, significant value could still be achieved from quality-controlled, properly timed and regularly repeated IRS using highly toxic insecticides, against which local vectors are proven to be both behaviourally and physiologically susceptible.

## Competing interests

While this study was independently funded primarily through the U.S. President’s Malaria Initiative via United States Agency for International Development (USAID), with other support awarded to SJM and FOO as stated above, one of the authors (SJM) also received support for the research projects from a manufacturer (Syngenta) of insecticidal public health products.

## Authors’ contributions

FO, JM, AN, DR and SJM designed and conducted the field and laboratory experiments, EM, GL, supervised the field experiments, FO, LL, MK, ET and SJM analysed the data. FO drafted the manuscript. All authors approved the final version of the manuscript.

## Supplementary Material

Additional file 1 Further details on proportions of dead mosquitoes caught in experimental huts: analysis of household level protection conferred by the different insecticidal interventions. Click here for file
