# Supplementary material for: Comparative field evaluation of combinations of long-lasting insecticide treated nets and indoor residual spraying, relative to either method alone, for malaria prevention in an area where the main vector is Anopheles arabiensis
Source: Parasit Vectors. 2013 Feb 22;6:46. doi: 10.1186/1756-3305-6-46 (PMC3606331; doi:10.1186/1756-3305-6-46)
Supplement: Additional file 1 — Further details on proportions of dead mosquitoes caught in experimental huts: analysis of household level protection conferred by the different insecticidal interventions. [file 1756-3305-6-46-S1.doc]

**Additional file 1**

### *Further details on proportions of dead mosquitoes caught in experimental huts: analysis of household level protection conferred by the different insecticidal interventions*

The mortality of mosquitoes caught in experimental huts with various treatments are shown in Table S1 and S2

*First spray round:* All IRS treatments, all LLINs and their combinations significantly increased proportion of dead *An. arabiensis* relative to controls. The most toxic IRS relative to controls was pirimiphos methyl (RR = 2.200 (1.734 – 2.792), P < 0.001), followed by lambda cyhalothrin (RR = 1.920 (1.582 – 2.330), P < 0.001) then DDT as the least toxic IRS (RR = 1.730 (1.398 – 2.140), P < 0.001). Of the LLINs, PermaNet 2.0® were the most toxic relative to controls (RR = 2.300 (1.981 – 2.672), P < 0.001), followed by Icon Life®­ (RR = 2.177 (1.914 – 2.477), P < 0.001) the Olyset® nets as the least toxic to malaria vectors (RR = 1.315 (1.172 – 1.590), P < 0.001).

Regarding addition of IRS onto LLINs (Figure 1), data from the first round showed that there was mostly no statistically significant increase of proportional mortality among malaria vectors caught in huts having LLINs plus IRS, relative to huts having LLINs alone, except where IRS treatment was pirimiphos-methyl. Where Olyset® nets were considered the baseline intervention, adding pirimiphos methyl IRS significantly increased proportional mortality of *An. arabiensis* relative to Olyset® nets alone (RR = 2.218 (1.194 – 4.118), P = 0.012), but there was no such effect with DDT (RR = 0.940 (0.510 – 1.732), P = 0.843) or lambda cyhalothrin (RR = 1.717 (0.965 – 3.055), P = 0.538). Similarly, where PermaNet 2.0® were used, pirimiphos methyl significantly increased percentage mortality of *An. arabiensis* (RR = 2.264 (1.218 – 4.207), P = 0.010) but there was no such effect with DDT (RR = 0.920 (0.524 – 1.797), P = 0.924) or lambda cyhalothrin (RR = 1.313 (0.734 – 2.349), P = 0.359). Finally, where Icon Life® nets were considered baseline intervention, IRS with pirimiphos methyl (RR = 1.401 (1.169 – 1.680), P < 0.001), but not lambda cyhalothrin (RR = 1.093 (0.922 – 1.295), P = 0.306) increased percentage mortality of *An. arabiensis* relative to the LLINs alone. In this situation however, IRS with DDT actually reduced proportional mortality of *An. arabiensis* relative to Icon Life® alone (RR = 0.767 (0.636 – 0.925), P = 0.006).

Regarding addition of LLINs onto IRS, we observed in the first round that unlike in cases of adding IRS onto LLINs, there were mostly statistically significant increases in proportional mortality among malaria vectors caught in huts with IRS plus LLINs relative to mortality in huts with IRS alone, except where the specific LLINs added were Olyset®, which had no such effects, and where the IRS was with DDT, which also seemed to hinder such additional effects. In huts sprayed with pirimiphos methyl, there was a significant increase in *An. arabiensis* mortality whenever volunteers sleeping in these huts used Icon Life® (RR = 1.387 (1.224 – 1.571), P < 0.001) or PermaNet 2.0® (RR = 1.773 (1.544 – 2.036), P < 0.001), but not Olyset® nets (RR = 0.994 (0.866 – 1.141), P = 0.930), relative to the IRS alone. Similarly in huts sprayed with lambda cyhalothrin, *An. arabiensis* mortality significantly increased when volunteers sleeping in these huts used Icon Life® nets (RR = 1.239 (1.094 – 1.405), P < 0.001) or PermaNet 2.0® (RR = 1.461 (1.285 – 1.662), P < 0.001), but not Olyset®, which instead decreased the proportional mortality relative to lambda cyhalothrin alone (RR = 0.841 (0.739 – 0.956), P = 0.008). On the contrary, in DDT-sprayed huts, no LLINs increased or decreased proportional mortality of *An. arabiensis* relative to DDT alone, except for a marginal increase when PermaNet 2.0® were used (RR = 1.184 (0.998 – 1.404), P = 0.052).

*Second spray round:* Results from this round were generally similar to results from the first round, with regard to proportional mortality of malaria vectors. All IRS treatments, LLINs and LLIN-IRS combinations significantly increased proportion of dead *An. arabiensis* mosquitoes, relative to the controls. Like in the first round, the most toxic IRS, relative to the control was pirimiphos methyl (RR = 2.208 (1.821 – 2.677), P < 0.001), followed by lambda cyhalothrin (RR = 1.551 (1.274 – 1.887), P < 0.001), then DDT (RR = 1.444 (1.181 – 1.766), P < 0.001). Besides, the most toxic LLINs relative to controls were again PermaNet 2.0® (RR = 1.654 (1.575 – 1.736), P < 0.001), followed by Icon Life® (RR = 1.545 (1.415 – 1.688), P < 0.001), then Olyset® nets (RR = 1.328 (1.119 – 1.470), P < 0.001).

Regarding addition of IRS onto LLINs (Figure 1), analysis of the second round data revealed that in most cases, there was no increase of malaria vector mortality in huts having LLINs plus IRS, relative to huts having LLINs alone, except where the specific IRS treatment was pirimiphos-methyl. Where Olyset® nets were considered baseline intervention, adding IRS with pirimiphos methyl significantly increased proportional mortality of *An. arabiensis* (RR = 1.375 (1.143 – 1.654), P = 0.001), but there was no such effect with DDT (RR = 1.174 (0.968 – 1.425), P = 0.103), or lambda cyhalothrin (RR = 1.075 (0.889 – 1.301), P = 0.455). Similarly, PermaNet 2.0® nets were used, adding IRS with pirimiphos methyl significantly increased proportional mortality of *An. arabiensis* (RR = 1.420 (1.179 – 1.710), P <0.001), but again there was no such effect with DDT (RR = 1.031 (0.849 – 1.253), P = 0.756), or lambda cyhalothrin (RR = 1.173 (0.969 – 1.419), P = 0.103). The results were similar when starting with Icon Life® nets, as percentage mortality of *An. arabiensis* significantly increased with addition of pirimiphos-methyl (RR = 1.237 (1.029 – 1.486), P = 0.023), but not DDT (RR = 0.995 (0.821 – 1.205), P = 0.958) or lambda cyhalothrin (RR = 1.008 (0.835 – 1.217), P = 0.972). The mortality-related effects of IRS when added to nets in rounds I and II are summarised in Figure 1.

Regarding addition of LLINs onto IRS, we observed again in the second spray round that proportional mortality of malaria vectors was significantly higher in huts having IRS plus LLINs, relative to huts having just IRS alone, except in certain cases where the LLINs added were Olyset® nets and where the IRS was based on DDT. In huts sprayed with pirimiphos methyl, proportional mortality of *An. arabiensis* was significantly higher when Icon Life® nets (RR = 1.386 (1.182 – 1.626), P < 0.001), PermaNet 2.0® nets (RR = 1.262 (1.075 – 1.481), P = 0.004) and even Olyset® (RR = 1.321 (1.126 – 1.549), P = 0.001) were added, relative to huts with pirimiphos methyl IRS alone. In huts sprayed with lambda cyhalothrin, there was a significant increase in proportional mortality of *An. arabiensis* whenever volunteers sleeping in these huts used Icon Life® (RR = 1.433 (1.190 – 1.725), P < 0.001) or PermaNet 2.0® (RR = 1.698 (1.354 – 2.129), P < 0.001), but not Olyset® nets (RR = 1.157 (0.921 – 1.454), P = 0.210), relative to just the IRS alone. On the contrary, we observed just like in the first spray round that in DDT sprayed huts, none of the LLINs affected proportional mortality of *An. arabiensis* mosquitoes, except PermaNet 2.0® nets which increased the mortality relative to the IRS alone (RR = 1.181 (1.056 – 1.320), P = 0.003).

Though toxicity of all treatments to *Culex* mosquitoes was evidently lower than toxicity to *An. arabiensis*, data from both rounds show that relative to controls, higher proportions of *Culex* were killed in huts sprayed with pirimiphos methyl or lambda cyhalothrin (P ≤0.003) and in huts having DDT coupled with PermaNet 2.0® nets (P < 0.001). Also, higher proportions of *Mansonia* mosquitoes died in huts with DDT coupled with PermaNet 2.0® in the first round (P = 0.001). Similarly in second round, all treatments except DDT alone (P = 0.245) or DDT coupled with Icon Life® nets (P = 0.374) killed significantly higher *Mansonia* proportions than controls.

### *Further details on actual number of mosquitoes killed per night in different huts: analysis of communal protection conferred by the different insecticidal interventions*

In addition to computing the proportional mortality among mosquitoes that entered different experimental huts, we also analysed and compared the actual numbers of mosquitoes killed per hut per night, regardless of the total numbers of mosquitoes actually entering the huts. Unlike proportional mortality which is useful for estimating household level protection conferred to users, the actual total number of mosquitoes killed by any intervention can indicate the extent of mass communal protection achievable from that intervention; since by killing potentially infectious vectors, these mosquitoes are actually prevented from ever transmitting disease to any other community members, including members of other households. In addition to the percentage mortalities, Tables S1 and S2 therefore also show actual totals of *Anopheles arabiensis,* *Culex* and *Mansonia* mosquitoes killed by different interventions.

*First spray round:* All the treatments and their combinations increased actual numbers of *An. arabiensis* mosquitoes killed per hut per night, relative to controls except DDT when used alone (P = 0.311) or in combination with Olyset® nets (P = 0.063). Of the IRS treatments, pirimiphos methyl killed the highest number of malaria vectors per hut per night relative to controls (RR = 2.889 (1.751 – 4.767), P < 0.001), followed by lambda cyhalothrin (RR = 2.112 (1.295 – 3.446), P = 0.003), then DDT, which killed the fewest relative to controls (RR = 1.297 (0.785 – 2.142), P = 0.311). Of the LLINs, Icon Life® nets killed the highest number of *An. arabiensis* relative to controls (RR = 2.620 (2.162 – 3.176), P < 0.001), followed by PermaNet 2.0® (RR = 1.975 (1.573 – 2.480), P < 0.001), then Olyset® nets (RR = 1.628 (1.297 – 2.044), P < 0.001).

Regarding addition of IRS onto LLINs in the first round, there were no increases in numbers of malaria vectors killed nightly in huts having LLINs plus IRS, relative to huts with LLINs alone, except where the IRS treatment was pirimiphos-methyl. Analysis showed that pirimiphos methyl actually doubled the number of *An. arabiensis* killed per night in huts having Olyset® nets (RR = 2.397 (1.463 – 3.928), P <0.001) or huts having PermaNet 2.0® nets (RR = 2.184 (1.337 – 3.566), P = 0.002). This IRS also significantly increased counts of dead *An. arabiensis* when it was added onto Icon Life® nets (RR = 1.735 (1.064 – 2.830), P = 0.027). On the contrary, DDT and lambda cyhalothrin did not have such effects on any of three LLINs (P > 0.05).

Regarding addition of LLINs onto IRS, we observed that unlike in the case of adding IRS onto LLINs, but similar to findings on proportional mortality, there were mostly significant increases in numbers of malaria vectors killed nightly in huts with IRS plus LLINs, relative to huts with just IRS, except in a few cases where the LLINs were Olyset® or where IRS treatment was DDT. In huts sprayed with pirimiphos methyl, numbers of *An. arabiensis* killed per night significantly increased when volunteers in these huts used Icon Life® (RR = 1.532 (1.217 – 1.929), P < 0.001), PermaNet 2.0® (RR = 1.493 (1.170 – 1.905), P < 0.001) or Olyset® nets (RR = 1.351 (1.048 – 1.740), P = 0.020). In huts sprayed with lambda cyhalothrin, there was also a significant increase in counts of dead *An. arabiensis,* following addition of Icon Life® (RR = 1.747 (1.401 – 2.177), P < 0.001) and PermaNet 2.0® (RR = 2.056 (1.011 – 1.562), P = 040), but not Olyset® nets (RR = 1.777 (0.946 – 1.464), P = 0.143). However, in DDT huts, only Icon Life® nets significantly increased the dead *An. arabiensis* counts (RR = 1.504 (1.206 – 1.876), P < 0.001), and instead there was only a marginal increase on addition of PermaNet 2.0® nets (RR = 1.278 (0.991– 1.649), P = 0.059), and no effect at all when Olyset® nets were used.

*Second spray round:* Findings from this round, regarding actual numbers of malaria vectors killed per hut per night, were also similar to those obtained in the first round. All IRS treatments, all LLINs and their combinations significantly increased numbers of dead *An. arabiensis*, relative to controls. IRS with pirimiphos methyl killed the highest number of the vectors per hut per night, compared to controls (RR = 2.853 (2.231 – 3.647), P < 0.001), followed by lambda cyhalothrin (RR = 2.484 (1.919 – 3.215), P < 0.001), then DDT (RR = 1.608 (1.238 – 2.089), P < 0.001). Of the LLINs, Icon Life®­ killed the most *An. arabiensis* relative to controls (RR = 2.349 (2.027 – 2.723), P < 0.001), followed by PermaNet 2.0® (RR = 1.917 (1.604 – 2.192), P < 0.001), then Olyset® nets (RR = 1.832 (1.533 – 2.190), P < 0.001).

Regarding addition of IRS onto LLINs, data from the second spray round showed that IRS with pirimiphos methyl or lambda cyhalothrin (in some cases), but not DDT, significantly increased numbers of malaria vectors dying per night in huts with any of the LLINs. Relative to Olyset® nets used alone, the total numbers of dead *An. arabiensis* were significantly higher if there was also IRS with pirimiphos methyl (RR = 2.056 (1.622 – 2.608), P < 0.001) or lambda cyhalothrin (RR = 1.481 (1.151 – 1.905), P = 0.002), but not DDT (RR = 1.189 (0.923 – 1.532), P = 0.181). Similarly, relative to PermaNet 2.0® nets alone, pirimiphos methyl (RR = 1.877 (1.480 – 2.381), P < 0.001) or lambda cyhalothrin (RR = 1.540 (1.198 – 1.980), P = 0.001) but not DDT (RR = 1.098 (0.852 – 1.415), P = 0.470) increased the total counts of dead vectors. Lastly, only IRS with pirimiphos methyl (RR = 2.230 (1.527 – 3.255), P < 0.001) but not lambda cyhalothrin (P = 0.961) or DDT (P = 0.078) increased numbers of *An. arabiensis* mosquitoes killed nightly in huts where volunteers were using Icon Life® nets, compared to huts where the Icon Life® nets were used alone without IRS.

Regarding addition of LLINs onto IRS treatments, the total number of malaria vectors killed per night was mostly higher in huts having IRS plus LLINs, compared to huts with IRS alone, except in certain specific cases where the LLINs were Olyset® nets or where the IRS was based on DDT. In huts sprayed with pirimiphos methyl, there was a significant increase in the number of *An. arabiensis* mosquitoes killed per hut per night whenever Icon Life® nets (RR = 1.937 (1.361 – 2.756), P < 0.001), PermaNet 2.0® nets (RR = 1.484 (1.054 – 2.090), P = 0.024), or Olyset® nets (RR = 1.551 (1.101 – 2.184), P = 0.012) were added relative to the IRS was used with just untreated nets. In the huts sprayed with lambda cyhalothrin, there was also a significant increase in number of dead *An. arabiensis* whenever volunteers sleeping in these huts used Icon Life® nets (RR = 1.257 (1.075 – 1.420), P = 0.004), a marginal increase with PermaNet 2.0® (RR = 1.189 (0.986 – 1.434), P = 0.069), but no change at all when Olyset® nets were added (RR = 1.092 (0.906 – 1.317), P = 0.355), relative to the lambda cyhalothrin IRS alone. Just like in the first round, we also observed that in DDT sprayed huts, none of the LLINs increased or decreased numbers of *An. arabiensis* mosquitoes killed per hut per night relative to the IRS alone (P > 0.05).

**Table S1:** Median percentage mortality (and inter-quartile ranges (IQR)), and the sums of mosquitoes of different taxa killed per night in experimental huts fitted with different IRS and LLIN treatments during the **first spray round.**

|  | **Mortality of *Anopheles arabiensis*** | | **Mortality of *Culex* species** | | **Mortality of *Mansonia* species** | |
| --- | --- | --- | --- | --- | --- | --- |
| **IRS/LLIN combinations** | **Median % (IQR)** | **Total number killed (n)^** | **Median% (IQR)** | **Total number killed**$ | **Median% (IQR)** | **Total number killed**$ |
|
|  |  |  |  |  |  |  |
| Untreated nets only** | 07.1 (3.8 - 14.0) | 403 (60) | 1.0 (0.0 - 06.2) | 77 | 16.5 (5.5 - 36.9) | 170 |
| Olyset only | 11.8 (7.1 - 17.2) | 709 (60) | 3.9 (0.0 - 08.8) | 121 | 33.3 (6.2 - 50.0) | 285 |
| PermaNet only | 19.5 (13.6 - 26.5) | 844 (60) | 2.4 (0.0 - 09.0) | 87 | 50.0 (39.6 - 70.1) | 343 |
| Icon Life only | 19.0 (12.4 - 27.5) | 1028 (60) | 2.7 (0.0 - 11.1) | 111 | 50.0 (29.6 - 62.8) | 444 |
|  |  |  |  |  |  |  |
| Actellic and untreated nets | 16.6 (12.1 - 28.7) | 836 (40) | 9.8 (2.6 - 20.4) | 136 | 42.9 (20.4 - 51.1) | 300 |
| Actellic and Olyset | 16.4 (13.1 - 24.9) | 980 (40) | 7.4 (2.3 - 16.7) | 102 | 41.2 (22.2 - 68.0) | 255 |
| Actellic and PermaNet | 29.0 (18.8 - 36.2) | 1196 (40) | 6.9 (2.3 - 15.3) | 98 | 71.8 (53.3 - 79.1) | 433 |
| Actellic and Icon Life | 21.0 (13.3 - 32.2) | 1338 (40) | 3.3 (0.3 - 12.5) | 108 | 56.5 (36.6 - 70.3) | 433 |
|  |  |  |  |  |  |  |
| DDT and untreated nets | 14.0 (07.7 - 24.4) | 369 (40) | 1.4 (0.0 - 13.3) | 52 | 50.0 (18.8 - 66.7) | 192 |
| DDT and Olyset | 13.2 (08.8 - 17.2) | 411 (40) | 3.0 (0.0 - 11.0) | 53 | 46.7 (21.1 - 62.4) | 162 |
| DDT and PermaNet | 17.2 (12.0 - 25.7) | 431 (40) | 4.2 (0.0 - 12.9) | 94 | 53.8 (36.7 - 66.7) | 220 |
| DDT and Icon Life | 12.3 (09.3 - 18.6) | 581 (40) | 1.8 (0.0 - 08.8) | 69 | 36.1 (20.2 - 50.0) | 165 |
|  |  |  |  |  |  |  |
| Lambda cyhalothrin and untreated nets | 14.8 (10.6 - 22.2) | 634 (40) | 6.3 (0.3 - 09.9) | 106 | 50.0 (25.0 - 66.9) | 304 |
| Lambda cyhalothrin and Olyset | 14.9 (09.6 - 20.6) | 755 (40) | 6.8 (2.0 - 17.7) | 98 | 66.7 (42.9 - 91.6) | 232 |
| Lambda cyhalothrin and PermaNet | 20.6 (15.3 - 26.5) | 802 (40) | 6.3 (0.3 - 13.6) | 110 | 64.3 (50.0 - 80.0) | 307 |
| Lambda cyhalothrin and Icon Life | 21.6 (16.8 - 26.9) | 1055 (40) | 5.1 (1.4 - 18.9) | 114 | 62.7 (46.6 - 77.6) | 364 |

**^** The term ‘n’ refers to total number of replicates

$ The number of replicates (n) was the same as for *Anopheles arabiensis*

**Controls refer to unsprayed huts in which volunteer used untreated nets

**Table S2:** Median percentage mortality (and inter-quartile ranges (IQR)), and the sums of mosquitoes of different taxa killed per night in experimental huts fitted with different IRS and LLIN treatments during the **second spray round.**

|  | **Mortality of *Anopheles arabiensis*** | | **Mortality of *Culex*** | | **Mortality of *Mansonia* species** | |
| --- | --- | --- | --- | --- | --- | --- |
| **IRS/LLIN combinations** | **Median % (IQR)** | **Total number killed (n)^** | **Median% (IQR)** | **Total number killed**$ | **Median% (IQR)** | **Total number killed**$ |
|
|  |  |  |  |  |  |  |
| Untreated nets only** | 10.4 (04.2 - 18.1) | 968 (90) | 3.3 (0.0 - 10.0) | 137 | 0.0 (0.0 - 27.1) | 85 |
| Olyset only | 14.8 (09.3 - 23.9) | 1742 (90) | 2.9 (0.0 - 10.0) | 128 | 0.0 (0.0 - 42.5) | 86 |
| PermaNet only | 19.7 (11.2 - 30.1) | 1644 (90) | 3.8 (0.0 - 13.7) | 177 | 26.1 (0.0 - 50.0) | 147 |
| Icon Life only | 16.7 (07.2 - 26.4) | 2121 (90) | 2.3 (0.0 - 11.5) | 187 | 20.0 (0.0 - 46.6) | 198 |
|  |  |  |  |  |  |  |
| Actellic and untreated nets | 23.4 (12.9 - 36.7) | 1599 (60) | 5.7 (2.5 - 31.8) | 272 | 21.1 (03.9 - 50.0) | 119 |
| Actellic and Olyset | 20.3 (12.4 - 31.2) | 2171 (60) | 7.1 (3.6 - 21.0) | 291 | 31.7 (12.7 - 56.2) | 149 |
| Actellic and PermaNet | 25.0 (14.6 - 36.9) | 2146 (60) | 9.7 (4.1 - 28.6) | 284 | 50.0 (29.4 - 97.7) | 262 |
| Actellic and Icon Life | 21.8 (11.9 - 34.2) | 2305 (60) | 9.6 (3.8 - 33.6) | 316 | 45.0 (28.6 - 79.5) | 282 |
|  |  |  |  |  |  |  |
| DDT and untreated nets | 17.1 (08.0 - 28.3) | 943 (60) | 3.6 (0.0 - 14.0) | 109 | 8.0 (0.0 - 38.3) | 68 |
| DDT and Olyset | 19.2 (11.6 - 28.1) | 1201 (60) | 4.3 (0.0 - 11.1) | 124 | 22.5 (0.0 - 50.0) | 65 |
| DDT and PermaNet | 19.4 (12.6 - 34.1) | 1171 (60) | 4.8 (0.0 - 24.3) | 150 | 33.3 (0.0 - 66.7) | 97 |
| DDT and Icon Life | 14.7 (09.7 - 24.1) | 1255 (60) | 4.6 (0.0 - 10.6) | 151 | 1.5 (0.0 - 30.6) | 60 |
|  |  |  |  |  |  |  |
| Lambda cyhalothrin and untreated nets | 17.8 (10.4 - 28.6) | 1431 (60) | 9.7 (4.9 - 22.5) | 197 | 21.1 (9.2 - 45.7) | 138 |
| Lambda cyhalothrin and Olyset | 14.2 (09.0 - 27.7) | 1578 (60) | 5.5 (0.0 - 15.4) | 157 | 25.0 (0.0 - 50.0) | 136 |
| Lambda cyhalothrin and PermaNet | 19.0 (10.8 - 33.4) | 1768 (60) | 7.7 (2.6 - 23.6) | 189 | 50.0 (8.5 - 80.0) | 264 |
| Lambda cyhalothrin and Icon Life | 18.4 (09.3 - 26.2) | 1893 (60) | 8.0 (1.5 - 16.9) | 155 | 33.3 (16.2 - 50.0) | 210 |

**^** The term ‘n’ refers to total number of replicates

$ The number of replicates (n) was the same as for *Anopheles arabiensis*

**Controls refer to unsprayed huts in which volunteer used untreated nets
